# Rhythmic activity in resting-state EEG predicts trait anxiety

**DOI:** 10.1162/IMAG.a.44

**Published:** 2025-06-18

**Authors:** Tamari Shalamberidze, Kyle Nash, Jeremy B. Caplan

**Affiliations:** Neuroscience and Mental Health Institute, University of Alberta, Edmonton, AB, Canada; Department of Psychology, University of Alberta, Edmonton, AB, Canada

**Keywords:** resting EEG, oscillations, theta, alpha, anxiety, BIS

## Abstract

Trait anxiety is characterized by heightened apprehension and worry about future events, with individuals experiencing it at varying levels. Identifying physiological markers of these differences could enhance our understanding of anxiety-related processes. We tested the hypothesis that resting-state rhythmic electroencephalography (EEG) activity (oscillations during alternating 1-minute eyes-open/eyes-closed rest conditions) predicts trait anxiety, as measured by the Behavioral Inhibition Scale (BIS). Multiple regression revealed that during eyes-open rest, higher frontal midline theta and lower frontal midline alpha activity significantly predicted BIS scores. A more stringent analysis confirmed that frontal midline theta and alpha activity met stricter criteria for rhythmicity. Interestingly, only parietal midline alpha power during eyes-open rest, but not oscillations, was predictive of BIS, suggesting that rhythmicity may differentiate the functional roles of frontal and posterior alpha in relation to anxiety. We also found that alpha asymmetry (power or oscillations) did not predict BIS scores. Our results suggest that heightened frontal midline theta during eyes-open rest may reflect increased baseline vigilance in individuals with high trait anxiety. Although speculative, lower frontal midline alpha may indicate greater cortical activation in regions involved in inhibition and cognitive control. Moreover, by differentiating power and oscillation effects, our findings help resolve ambiguities in related research.

## 1 Introduction

Trait anxiety is associated with a tendency toward increased apprehension and worry about future events (Barlow, 2004), which, in turn, can influence emotional and cognitive processes. There is accumulating evidence that some of these effects are driven by rhythmic brain activity. Here we focus on brain activity during rest (no task), when we expect anxiety-related apprehensive and worrying thoughts will be prominent. As we summarize below, evidence linking resting rhythmic neural activity to trait anxiety remains mixed, likely due to methodological differences in signal analysis, such as measures of rhythmic (oscillatory) activity also being strongly influenced by non-rhythmic activity, reducing the specificity of the measure, as well as variations in how the rest state is induced. In this study, we focus more directly on rhythmic activity (while also comparing it with the more conventional, less-selective power measure) during a rest state that is designed to plausibly allow anxiety-related thoughts and emotions to emerge.

**Theories of anxiety.**According to reinforcement sensitivity theory, two primary systems regulate emotion and behavior: the Behavioral Inhibition System (BIS) and the Behavioral Activation System (BAS;Gray, 1982). BIS manages motivational conflict by interrupting behavior in uncertain or risky situations, driving anxiety and prompting risk assessment. It heightens vigilance in response to perceived threats, especially when conflicting motivations, such as approach and avoidance, are present. Unlike the Fight-Flight-Freeze System (FFFS), which handles immediate fear responses to clear danger, the BIS governs anxiety-driven caution and deliberation (Gray & McNaughton, 2000). In contrast, the BAS drives approach behaviors, motivating individuals to pursue goals or rewards.

Individuals with higher levels of trait anxiety often exhibit an overactive BIS, leading to excessive behavioral inhibition and an increased likelihood of avoiding goal-directed behaviors to prevent possible adverse outcomes, such as declining promising opportunities due to fear of failure (Gray, 1982;Gray & McNaughton, 2000). Moreover, high BIS levels have been associated with greater trait anxiety and neuroticism (Campbell-Sills et al., 2004;Carver & White, 1994;Smits & Boeck, 2006). Although anxiety serves a necessary evolutionary purpose and behavioral inhibition is essential for appropriate functioning, an overactive BIS and elevated trait anxiety can become maladaptive, significantly impacting daily life. Therefore, identifying the neural correlates of BIS is crucial for advancing our understanding of anxiety-related processes and dysfunctions.

Despite the theoretical strength of BIS models, the connection between animal-based frameworks and human trait anxiety remains unclear. This study addresses these gaps by focusing on rhythmic neural activity, isolating its contributions from non-rhythmic sources, and refining the neural signals associated with trait anxiety. Our findings demonstrate that neural rhythms provide valuable insights into stable personality traits, advancing understanding of how intrinsic brain activity shapes individual differences in emotion, cognition, and behavior. Additionally, this work enhances methodologies for studying resting-state neural rhythms, with broader applications for exploring other personality dimensions and emotional traits influenced by similar neural mechanisms.

**The value of investigating resting rhythmic brain activity related to anxiety.**Electroencephalography (EEG) is commonly used to measure such neural correlates of anxiety, including rhythmic activity. Much of the existing research has focused on the connection between trait anxiety and neural activity during specific tasks or behaviors. In contrast, resting-state EEG activity, also viewed as a neural trait, reflects relatively stable neural patterns that are independent of task engagement (Näpflin et al., 2007;Nash et al., 2014). As such, linking trait anxiety to resting-state EEG may provide deeper insights into intrinsic anxiety-related neural processes, unaffected by the external demands of tasks or stimuli but potentially more influenced by internalized processes, such as intrusive thoughts, rumination, and worry.

The nature of the resting state remains debated, with some suggesting it represents mere “idling,” while others argue it involves complex cognitive processes such as daydreaming and rumination (Gusnard et al., 2001;Menon, 2023;Pfurtscheller, 1992). Evidence also suggests that sleep may be common during resting states, especially if they extend beyond 3 minutes (Tagliazucchi & Laufs, 2014). This debate highlights that the resting state is not homogenous; for some individuals, it may predominantly reflect stable, low-arousal neural activity, whereas for others, especially those prone to anxiety, it may involve more active cognitive processes, such as rumination. This variability suggests that mental traits can uniquely influence the nature of rest and how it is reflected in EEG recordings. Considering the high test–retest reliability of the resting-state EEG (Metzen et al., 2022;Nash et al., 2014), when recorded under 3 minutes, it becomes a valuable tool for assessing individual differences in the neural correlates of personality traits.

**Methods of quantifying rhythmic activity, limitations, and solutions.**Conventional spectral analysis measures total power which is known to combine both rhythmic (oscillatory) and non-rhythmic sources, collapsing these different processes together (He, 2014;Klimesch, 1999;Whitten et al., 2011). Distinguishing between rhythmic and non-rhythmic power sources is crucial as they reflect different physiological processes: rhythms indicate synchronized neural firing and network coordination, while non-rhythmic power can include transient events, or other non-oscillatory neural activity. To address these issues, we use the Better OSCillation detection (BOSC) method, which calibrates measures based on the signal’s background spectrum (“colored noise”) and applies additional thresholds for rhythmicity (Caplan et al., 2001). Importantly, this distinction may help clarify discrepancies in the literature, as combining rhythmic and non-rhythmic signals without distinguishing their nature could obscure the specific contributions of each to anxiety-related neural activity. Therefore, separating these signal types allows us to more accurately identify neural oscillations linked to behavioral inhibition and anxiety regulation, providing insights that may be overlooked when only total power is considered.

### 1.1 Resting-state alpha

Alpha (8–12 Hz) is a dominant rhythm in adult EEG and is most often and easily detected during rest (Berger, 1929). Traditionally, alpha power was believed to be inversely related to cortical activation in adults (Davidson, 1992). This idea was initially supported by the observation that, at posterior electrodes, alpha power is higher when individuals close their eyes (reduced visual cortex activation), and lower when their eyes are open (increased visual cortex activation). Further research confirmed that alpha activity is also suppressed by other, non-visual mental processes (Allen et al., 2004). This led to the hypothesis that different cortical areas exhibit their own alpha rhythms, representing a resting or “idling” state of those brain regions (Kropotov, 2009;Palva & Palva, 2007).

Beyond single-electrode analysis, researchers have also investigated global alpha (across-electrode mean activity) measurements. Consistent with the “idling” hypothesis, transitioning from a less aroused Eyes Closed (EC) condition to a more aroused Eyes Open (EO) condition decreases global alpha power (Barry et al., 2007). Evidently, this change is accompanied by increased skin conductance levels, a well-established physiological marker of arousal and stress. The negative correlation between EC resting global alpha power and skin conductance further supports the view of alpha activity as an inverse marker of arousal (Barry et al., 2020). Collectively, these studies suggest that higher alpha power is associated with idling or inactive states, whereas lower alpha power corresponds to more active, aroused states, which may also be linked to higher anxiety.

Surprisingly, a contrasting body of research indicates that higher global alpha power, both during rest and task presentation, is positively associated with trait anxiety (measured by Taylor Manifest Anxiety and State and Trait Anxiety Inventory, STAI) and behavioral inhibition (measured by the Gray–Wilson Personality Questionnaire;Knyazev et al., 2002,^2003^,^2004^). Overall, Knyazev and colleagues found that higher frequencies, such as alpha, were predominant in individuals with higher BIS scores, and lower frequencies (theta and below) were more prevalent in individuals with higher BAS scores. Notably, their analysis sometimes combined global mean alpha power across both eyes-open and eyes-closed resting states (Knyazev et al., 2003), or included only eyes-closed rest (Knyazev et al., 2004). This contrasts with studies that focus on specific resting states (eyes open vs. eyes closed), which are considered functionally independent, potentially introducing ambiguity in interpreting the results.

***Resting-state frontal alpha asymmetry***(right hemisphere alpha activity compared with left), and its association with affect and emotion, is another contentious subject. Evidence from decades of research suggests that frontal cortical asymmetry explains a large amount of individual variability in emotional behavior and affective style, or personality traits (Davidson, 1992;Sutton & Davidson, 1997). In line with this, earlier studies demonstrated that the right prefrontal cortex is a biological substrate of withdrawal behavior and negative affect, and higher BIS is related to greater left frontal alpha power compared with right frontal alpha power (Sutton & Davidson, 1997). In contrast,Harmon-Jones and Allen (1997)did not find BIS to be related to the greater left frontal alpha, andCoan and Allen (2003)found only a weak statistical trend at neighboring electrodes. Moreover, a recent review byFirth et al. (2024)concluded that findings about BIS and frontal alpha asymmetry are largely inconsistent, and mainly absent during rest. (See alsoKołodziej et al. (2021), who found a similar null relationship between alpha asymmetry and depression.)

This inconsistency can be partially attributed to the complexity of the BIS system and its relationship with withdrawal behavior (Coan & Allen, 2003). Notably, Gray’s original Behavioral Inhibition System (Gray, 1982) was later divided into two distinct systems, the anxiety-related BIS and the fear-related Fight-Flight-Freeze System (FFFS) (Gray & McNaughton, 2000). Given that this distinction was not reflected in behavioral inhibition measuring scales, if withdrawal tendencies activating right frontal brain are triggered exclusively by threat cues and not anxiety, only certain aspects of the BIS scales might be linked to increased right frontal neural activity (Coan & Allen, 2003).

Although considerable attention has been given to posterior and global alpha, as well as frontal alpha asymmetry, frontal midline alpha power remains largely unexplored in relation to rest and its connection to anxiety and the behavioral inhibition system. Given the importance of anterior brain regions in negative emotions and anxiety, it is possible that beyond asymmetry, significant relationships may also emerge in midline electrodes (Gray & McNaughton, 2000).

The focus on the difference-based frontal asymmetry over single-electrode measures may stem not only from theoretical assumptions but also from methodological challenges. Consider, for example, that brain activity is usually referenced to a control condition, often a pre-stimulus rest state. If rest is the condition of interest, there is no obvious control to subtract out. This poses a difficulty interpreting resting-state EEG signal without comparison with a reference state. It also means there is no calibration of the measurement scale across participants, which limits removing large sources of subject variability simply due to measurement scale (which could be due to numerous effects of no interest, such as skull thickness). As a difference measure, asymmetry is thus appealing because the opposite hemisphere functions mathematically similarly to a control condition, providing some calibration. We speculate that because interpreting absolute power measurements is challenging without baseline values, this may have led researchers to favor asymmetry measures that essentially self-calibrate through comparisons between left and right hemispheric activity. However, we can now address the calibration problem with a method such as BOSC, which uses the signal’s own statistical properties to tune oscillatory detection thresholds (Caplan et al., 2001). This approach makes the oscillation measures comparable across participants and interpretable without additional mental-state reference. Importantly, this approach reveals how midline alpha activity contributes to anxiety and the behavioral inhibition system, complementing the insights gained from global and asymmetry measures.

### 1.2 Resting-state theta

Generally, minimal to no theta rhythm is detected during short periods of rest in human EEG recordings (Caplan et al., 2015). This poses a significant challenge for studying resting-state theta oscillations, especially when compared with the well-documented hippocampal theta in animals or task-related cortical theta in humans. Consequently, most studies have focused on task-related theta activity in relation to BIS and anxiety, whereas few have explored the connection between resting-state theta and anxiety. Yet, despite its apparent absence in most individuals, if the theta rhythm is not universally present but does legitimately occur in a subset of individuals, we may find that these sporadic theta oscillations reflect important individual differences. Our study aims to address this gap by investigating whether variability in resting-state theta could serve as a meaningful marker for anxiety-related processes.

On the other hand, the link between task-related theta activity and the behavioral inhibition system has been extensively studied, and the theoretical view of task-related theta activity might also extend to rest. The task-related theta rhythm was first investigated to understand the neuropsychology of anxiety in animal models (Gray, 1982). It has been suggested that theta power in the rodent hippocampus (over a more generous range, 4–12 Hz) is a neural hallmark of BIS and anxiety processes (Gray, 1982;Gray & McNaughton, 2000). The corresponding human model suggests that internal conflict between equally compelling but incompatible goals activates the septohippocampal system, which then resolves the goal conflict through recursive communication with the neocortex, facilitated by rhythmic activity in the theta range (Corr, 2008;Gray & McNaughton, 2000). Indeed, some studies indicate that goal conflict increases theta activity, aligning with the septohippocampal theory of anxiety (Cavanagh & Shackman, 2015;Moore et al., 2006;Neo et al., 2011;Shadli et al., 2021). However, these studies specifically relate to tasks activating goal conflict and behavioral inhibition, which may differ fundamentally from the concept of trait-like behavioral inhibition observed during rest without task engagement, as we investigate here. Additionally, variability in frequency ranges measured and the electrodes analyzed (elaborated in the discussion) introduces uncertainty about the precise role of theta rhythm in anxiety and BIS.

A few earlier studies related to theta activity and anxiety at rest offer results that seem to challenge BIS theory. For example,Mizuki et al. (1984,1989)observed that individuals with higher trait anxiety (as measured by Maudsley Personality Inventory and STAI) exhibit lower FMT (4.5–7.5 Hz) during a mental arithmetic task and do not exhibit FMT during rest. Interestingly, while FMT was absent at rest, it appeared in individuals after administering anxiolytic drugs, allowing authors to suggest that the appearance of FMT reflects relief from anxiety. Later,Suetsugi et al. (2000)confirmed these findings, reporting that non-medicated patients with generalized anxiety disorder, who initially lacked FMT, began to exhibit it following treatment, coinciding with symptom reduction. It is important to note that the EEG recordings in some of the earlier studies were captured on paper and analog tape recorders. Given the limitations of these older recording methods, it may be helpful to approach these findings and interpretations with some caution. In contrast, a sensor-level connectivity analyses have shown increased midline theta coherence during rest in individuals with generalized social anxiety disorder compared with healthy individuals (Xing et al., 2016).

Another opposing line of research, as discussed in the section on resting-state alpha, indicates that both at rest and during task presentation, BIS was associated with power at higher frequencies, such as alpha, whereas lower frequencies, such as theta, were linked to BAS (Knyazev et al., 2002,^2003^,^2004^). As already mentioned, the use of global theta measures and the combination of eyes-open and eyes-closed rest conditions in their analysis adds further ambiguity to BIS and theta, as well as alpha research.

Despite the controversy, the theoretical framework and most of the task-related research agree that the theta rhythm could be a valuable correlate of human anxiety, although the precise relationship, cortical region, or the frequency range of the rhythm remain unresolved.

### 1.3 Goals

It should be evident from this summary that the findings related to alpha and theta activity in anxiety research are highly variable and apparently contradictory. This inconsistency may stem from methodological differences (such as different questionnaires used to measure anxiety), as well as the diverse experimental and resting conditions employed. For instance, methodological limitations in earlier studies might explain the absence of FMT at rest, potentially influencing other contentious findings. In addition, the electrodes recorded and analyzed vary substantially. Some of the studies linking resting state and anxiety combine eyes-open and eyes-closed conditions in their analysis, leading to ambiguous results and interpretations.

Moreover, most research on the relationship between theta activity and BIS focuses on task-based, rather than resting-state, EEG. However, behavioral inhibition during tasks that trigger internal conflict may differ fundamentally from the concept of behavioral inhibition observed during rest, where no specific task demands are present. Therefore, gaining a comprehensive understanding of resting-state theta and alpha in anxiety could offer valuable insights not only into rest itself but also into how task-related activity relates to anxiety, serving as a reference point and refining our interpretation of these connections. Lastly, as discussed above, we suggest that one of the most significant factors contributing to inconsistent findings regarding both alpha and theta rhythms during rest may be the failure to distinguish between rhythmic oscillations and non-rhythmic components within spectral power (Caplan et al., 2015; see alsoKlimesch, 1999).

We address these inconsistencies in several ways. By applying a more rigorous method that reliably differentiates rhythmic from non-rhythmic power changes, we can directly test whether the power and oscillation measures match or contradict. By using a sufficiently long time period (1 minute), we aim to capture true rest, avoiding responses to rapid state transitions, but by keeping these shorter than many studies, we also avoid the onset of sleep. Moreover, to effectively measure anxiety during more and less aroused resting states, we analyze eyes-open and eyes-closed conditions separately. We also focus on theoretically grounded electrode selection. Considering that BIS and anxiety are primarily associated with anterior brain regions, we aim to address the current gap in understanding how resting-state frontal midline alpha and theta power, as well as oscillations, relate to BIS and trait anxiety. We hypothesize that the frontal midline alpha oscillations, and even the rare frontal midline theta oscillations detected at rest, might account for individual differences in trait anxiety.

In sum, we aim to (1) test the hypothesis that frontal midline alpha and theta oscillations predict BIS, (2) test the hypothesis that frontal alpha asymmetry predicts BIS, (3) compare the conventional power measurement with the oscillation measures, (4) test the hypothesis that posterior alpha, potentially related to alertness or visual inattention, also relates to BIS.

## 2 Methods

### 2.1 Design of the current study

The resting-state EEG experiment was part of a larger unpublished study investigating the role of neural oscillations in associative memory and anxiety. Resting-state EEG recording was performed after a brief break following the completion of associative memory task (not described here), anxiety and demographic questionnaires. To measure anxiety, we used Behavioral Inhibition and Activation Scale (BIS/BAS;Carver & White, 1994), State and Trait Anxiety Inventory (STAI;Spielberger et al., 1983), and Ten Item Personality Inventory (TIPI;Gosling et al., 2003).

### 2.2 Participants

Sixty-nine participant (all self-reported right-handed, 36 female) undergraduate students enrolled in an introductory psychology course at the University of Alberta, aged 17–51 years (mean = 20.51, SD = 4.86), participated for course credit. Data from 17 participants were excluded from analyses due to excessive amounts of artifacts in the EEG; because trial rejection was not possible considering the nature of resting-state recording, we removed participant data with more than 20% of bad electrodes or more than 20% of bad ICA components. An additional five participants’ data were excluded from BIS analysis due to incomplete BIS questionnaire data. We asked participants to report whether they had been diagnosed with anxiety or any other mental health disorders (10 out of 51 responded yes,∼20%). However, as we did not assess clinical anxiety ourselves, we did not use these self-reports in our analysis. Importantly, no participants were excluded from the analysis based on their responses.^*^Data from the remaining 51 participants for the STAI and TIPI and 46 participants for BIS were used for further analysis. All participants were required to either be fluent in English or have English as their first language (learned before the age of 6 years), have normal or corrected vision, and have normal or corrected hearing. Written informed consent was obtained prior to the experiment in accordance with a University of Alberta ethical review board.

### 2.3 Behavioral protocol

**Fig. 1.**
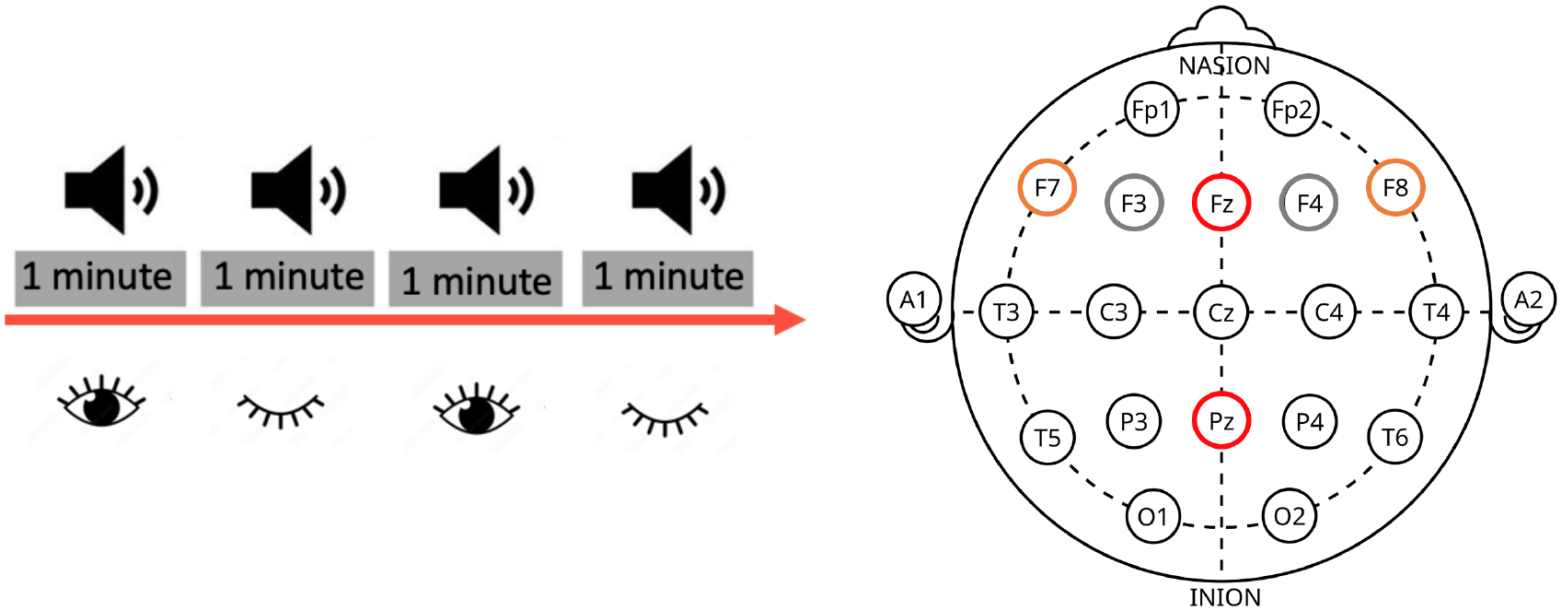
(Left) Experimental design: subjects received verbal and written instructions to begin with their eyes open and to close their eyes gently upon hearing a beep. The beep was presented every 1 minute (instructing participants to open or close their eyes) with four beep presentations in total, constituting 4 minutes (2 minutes eyes-open and 2 minutes eyes-closed) EEG recording. (Right) 21 electrodes of the International 10–20 system for EEG with highlighted electrodes were used in our analysis. Namely, red represents Fz and Pz electrodes used in alpha and theta power and oscillatory multiple regressions and correlations; gray represents F3 and F4 for mid-frontal alpha asymmetry, and orange represents F7 and F8 for lateral-frontal alpha asymmetry analyzes. Note: The EGI Geodesic-Sensor-Net used in the experiment consists of 256 electrodes; a 10–20 system is provided as an easier reference.

The session took place in an electrically shielded, sound-attenuated chamber. The task was presented in E-prime (Psychology Software Tools Inc., Pittsburgh, PA). After completing the associative recognition memory task, participants were given three anxiety questionnaires, followed by a short demographic questionnaire. After the completion of all the questionnaires, participants took a short break. After the break and receiving oral instructions, participants were shown the fixation cross and were given written instructions to keep their eyes open after the beep, until they heard the next beep (1-minute eyes-open condition), and then to close their eyes and open them only after they heard the next beep (1-minute eyes-closed condition). This was repeated twice, with four beep presentations in total, constituting 4 minutes of two 1-minute eyes-open and two 1-minute eyes-closed resting cycles (Fig. 1).

The BIS/BAS questionnaire consisted of 20 questions with a 7-point Likert scale. Seven questions assess BIS (anxiety), whereas the remaining items measure Behavioral Activation System subscales (BAS-Drive, BAS-Reward, and BAS-Fun Seeking). Therefore, we used only the seven anxiety-related items (items 2, 3, 6, 9, 12, 15, 18) representing a score range from 7 to 49 (mean: 37.5). Two items from BIS (12 and 18) were reverse scored. These reversed-scored items are also those that were scored as fear-related items when the BIS scale was divided into BIS-anxiety and BIS-FFFS measuring subscales. More precisely, according toJohnson et al. (2003), J-BIS (anxiety-measuring) subscale consists of items 2, 3, 6, 9, and 15, whereas J-FFFS (fear-measuring) subscale includes items 12 (even if something bad is about to happen to me, I rarely experience fear or nervousness) and 18 (I have very few fears compared with my friends).

The STAI consisted of 40 questions total, 20 for state and 20 for trait anxiety scales, with a 4-point Likert scale. We scored and used the trait anxiety scale, which represents a score range from 20 to 80 (mean: 51.7). The TIPI consisted of 10 questions total (2 for each personality type), with a 7-point Likert scale. We only scored and used two (items 4 and 9) questions measuring neuroticism. Item 9 was reverse scored. Respectively, the score ranges from 2 to 14 (mean: 8).

### 2.4 Electroencephalography (EEG) recording and analyses

EEG was recorded using a high-density 256-channel Geodesic Sensor Net (Electrical Geodesics Inc., a neurodiagnostic equipment company based in Eugene, OR), amplified at a gain of 1,000 and sampled at 500 Hz. Impedances were kept below 50 kΩ, and EEG was initially referenced to the vertex electrode (Cz). Participants were shown the recording artifacts produced by eye blinks, jaw clenching, limb movement, and other common tics to demonstrate the importance of remaining still. Data were analyzed by custom MATLAB scripts in conjunction with the open-source EEGLAB toolbox (Delorme & Makeig, 2004). Artifacts were corrected via Independent Component Analysis, implemented in EEGLAB (Jung et al., 2000), in conjunction with the ICLabel toolbox (Pion-Tonachini et al., 2019). This procedure classifies the components that capture muscle and eye-blink-related artifacts. The selection of components was based on visual inspection of spatial topographies, time courses, and power spectral characteristics of all components. ICLabel was used as an additional tool to classify eye, muscle, or heart components with a probability>80%, but the decision to exclude components was made after visually inspecting them and other suspicious components. The components accounting for stereotyped artifacts, including eye blinks, eye movements, and muscle movements, were removed from the data. Artifacts of line noise at 60 Hz were also removed. If more than 20% of ICs or electrodes needed to be removed, the subject data were not considered for further analysis. Event latencies were corrected with a time lag correction (18 ms) due to a known hardware calibration problem identified by EGI.

### 2.5 Oscillation analysis

The spectral analysis informs us about the different frequencies (cycle per second, Hz) and their power (measured in$\mu V^{2}\text{​}/\text{​}Hz$) at a given time window of the recording (Cohen, 2014). Conventional spectral analysis methods are not selective for rhythmicity of the signal. To test whether the neural activity is truly rhythmic or a non-rhythmic (non-repeating) signal, oscillations were detected and quantified using BOSC (Caplan et al., 2001;Whitten et al., 2011). The first step in the BOSC process is to calculate power using Morlet wavelet transform (Grossmann & Morlet, 1985). The subsequent steps involve determining the additional thresholds (explained below) to control for true rhythmicity. To check whether our results might generalize to more conventional spectral analysis methods, we ran parallel statistical analyses using BOSC and a measure of wavelet power (before additional thresholds are applied), which is expected to be less selective and relatively more sensitive to non-rhythmic signals. For clarity in terminology, we use “power” when referring to wavelet power and “oscillations” when referring to activity identified by BOSC after the thresholds were applied, passing its more stringent criteria for rhythmicity.

Both BOSC and power analysis were run on the continuous resting-state EEG recording. Our main interest for the single electrode analysis lies in frontal midline activity, thus the Fz is a primary electrode of interest. However, due to the alpha signal being stronger at the parietal locations and associated with changes in arousal, we decided to test the Pz electrode as well. For the frontal alpha asymmetry analysis, we selected two mid-frontal (F4–F3) and two lateral-frontal (F8–F7) electrode pairs. Thus, the spectral analysis was performed at five frontal (Fz, F3, F4, F7, F8) and one parietal (Pz) electrodes^†^(Fig. 1), using a Morlet wavelet transform (Grossmann & Morlet, 1985) with a window of 6 cycles and frequency sampled in 20 logarithmic steps covering the bandwidth from 1 to 32 Hz.

With BOSC analysis, the signals are only classified as rhythmic if they exceed a particular power threshold,${\text{P}}_{T}(f)$for a given frequency,f, and a particular duration threshold${\text{D}}_{T}(f)$for each frequency. To calculate the power threshold, the method takes into account and models the background “colored noise” spectrum of EEG. The colored noise spectrum is a basic property of EEG as well as other natural autocorrelated signals (Shlesinger & West, 1988). This spectrum is then fit with linear regression in log-log space. The power threshold (${\text{P}}_{T}$) is set to the$95^{th}$percentile of the theoretical probability distribution of power values at a given frequency, and the duration threshold (${\text{D}}_{T}$) is set at each frequencyfto three consecutive cycles (3​/​f). This ensures that detected oscillations are reliably different from the background noisy component of the EEG signal and does not include increases in spectral amplitude that are non-repeating. This method is thus more selective for truly rhythmic (repeating) activity than other methods (Caplan et al., 2001;Whitten et al., 2011).

The final measure derived from the BOSC method is termed$P_{episode}$, the proportion of time during which oscillations are detected at a given frequency. For example,$P_{episode}=0.8$means oscillations at the frequency of interest were detected during 80% of the recording (illustrated inFig. 2A). The frequency bands of interest were defined as theta, 4–8 Hz, and alpha, 9–14 Hz. The power and oscillation measures within a band were derived by averaging power or$P_{episode}$within that particular bandwidth, respectively.

**Fig. 2.**
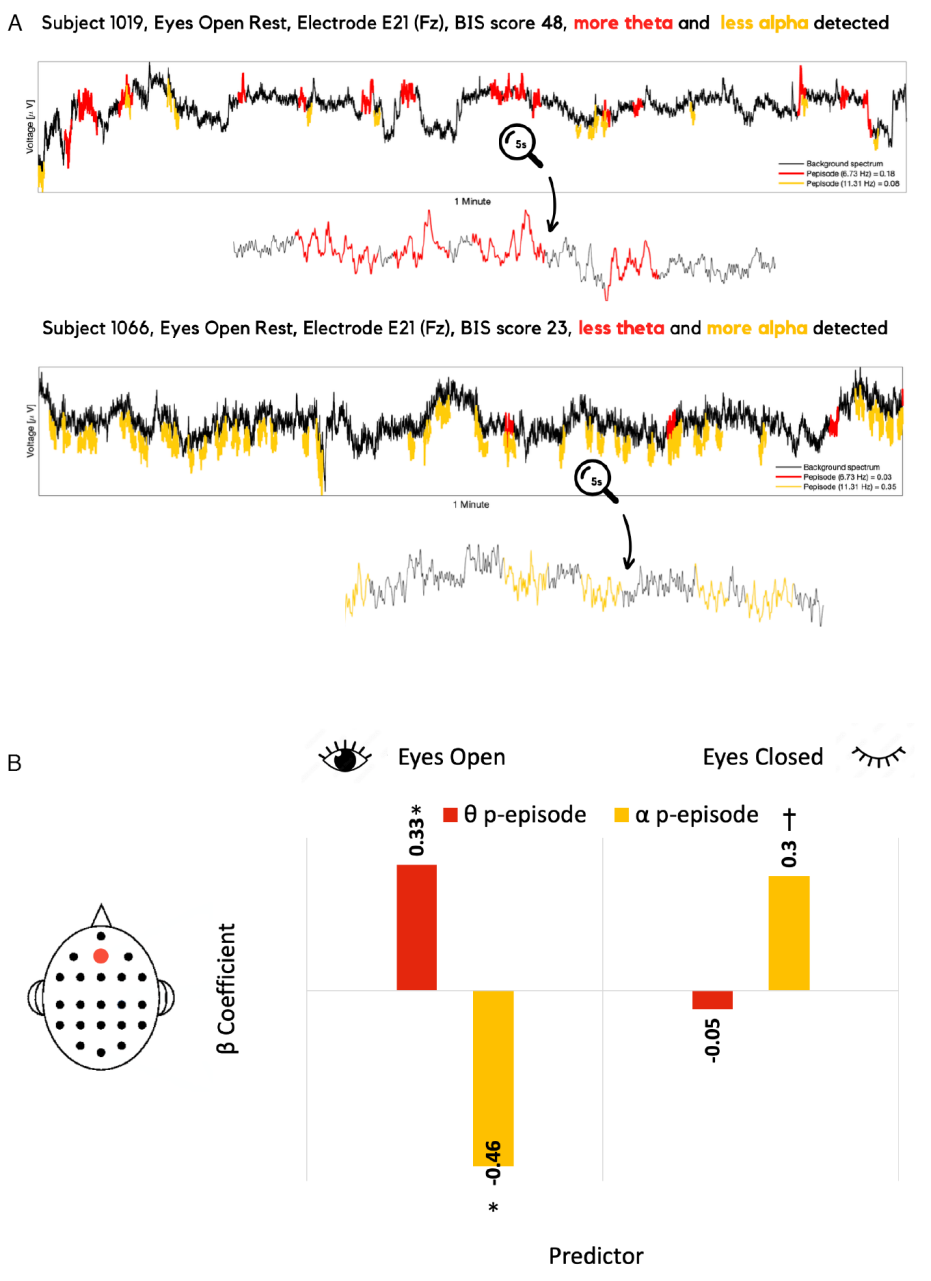
These figures demonstrate the detection of oscillatory activity (A) and its predictive relationship with BIS scores (B). (A) Example of the raw EEG traces recorded during eyes open rest at electrode Fz, with oscillations detected by BOSC in theta (in red) and alpha (in yellow) ranges in one high-BIS (top, score 48) and one low-BIS (bottom, score 23) participant. (B) The representation of the multiple regression model predicting BIS scores with theta and alpha oscillatory activity ($P_{episode}$) measures recorded at frontal midline (Fz) electrode during eyes open and eyes closed resting states. Red bars represent beta coefficients for theta oscillations predicting BIS, and yellow bars represent beta coefficients for alpha oscillations predicting BIS.

All trials were time locked to the onset of the beep presentation, and$P_{episode}$of all frequencies at each EO and EC trial was calculated using the time window of 1–60,000 ms latency post-stimulus, minus shoulders (12,000 ms) to avoid edge artifacts. This left 48,000 ms of each EO or EC phase. This shoulder subtraction also ensures that we are not measuring EEG activity right after the beep presentation to avoid the auditory signal processing and oculomotor processes (and artifact). Because the defining frequencies for a rhythm band (like theta and alpha) vary from study to study, it is also important to examine each frequency and its relationships with anxiety scores individually. To check the robustness of our frequency bands, we also examined our correlation analyses at all frequencies sampled over the 1–32 Hz range.

To measure frontal alpha asymmetry, the laterality quotient (LQ) was calculated by subtracting left frontal alpha power/oscillations from right frontal alpha power/oscillations—LQ =ln[R]−ln[L](Reznik & Allen, 2018). Thus, positive LQ values represent relatively higher right alpha power/oscillation (rightward asymmetry), whereas negative LQ values represent relatively higher left alpha power/oscillation (leftward asymmetry) (Metzen et al., 2022). If one assumes the inverse relationship between alpha power and cortical activity, positive LQ would reflect higher left cortical activity, whereas negative LQ would reflect higher right cortical activity. As already mentioned, we calculated alpha asymmetry at two pairs of electrodes, mid-frontal electrode pair (F4–F3) and lateral-frontal (F8–F7).

### 2.6 Statistical analysis

All statistical analyses were carried out using MATLAB and Statistic Toolbox Release 2020a (The MathWorks, Inc., Natick, Massachusetts, United States), and JASP (Jasp Team, University of Amsterdam, Netherlands) on power and$P_{episode}$for oscillations at the corresponding electrodes and time windows.

First, we conducted two multiple regression analyses, one at Fz and one at Pz, to explore whether BIS scores are predicted by alpha and theta oscillations during eyes-open and eyes-closed resting states. We then conducted two similar regressions (one at Fz and one at Pz) to test whether BIS scores are predicted by alpha and theta power during eyes-open and eyes-closed resting states. Next, we ran two additional regressions to test whether BIS scores are predicted by frontal alpha asymmetry (one with power and one with oscillations) at mid-frontal and lateral-frontal electrodes recorded during eyes-open and eyes-closed resting states. Finally, as an exploratory analysis, we conducted a post hoc regression combining Fz and Pz to test whether BIS scores are predicted by alpha and theta oscillations recorded during eyes-open and eyes-closed resting states, with a similar analysis for power measures. We combined Fz and Pz predictors in the regression to determine their unique contributions to predicting trait anxiety when considered simultaneously. We also conducted Pearson correlations between alpha and theta oscillations (and power) recorded at Fz and Pz electrodes during eyes open and eyes closed rest, and BIS, STAI and TIPI neuroticism scores. Correlations were computed both for the average values within each frequency band (theta and alpha) and for each individual sampled frequency (1 - 32 Hz). We also conducted a post hoc reliability analysis using bootstrapping with sub-sampled data segments ranging from 10% to 100% of the total duration.

Statistical tests are reported with both Classical and Bayesian approaches. Significance is assessed withα=0.05but p values near-threshold are interpreted with caution. Bayes Factors are considered to provide support for the null hypothesis if$BF_{inclusion}<1/3$or for the hypothesis if$BF_{inclusion}>3\text{​}/\text{​}1$(Kass & Raftery, 1995). All the BFs are reported in the brackets in the Results section and on tables, but only a subset is highlighted in the text.

## 3 Results

### 3.1 BIS and resting-state neural oscillation and power model at Fz

Multiple regression for BIS and resting-state oscillations (the BOSC measure) model at Fz electrode is summarized inTable 1and also visualized inFigure 2B. The model with four predictors (FzθEyes Open,θEyes Closed,αEyes Open,αEyes Closed) was significant, with adjusted$R^{2}=0.26$,$R^{2}=0.32$,F(4, 41)=4.88,p=0.003. Theθ(β=56.73,t(45)=2.51,p=0.016,$BF_{\text{inclusion}}=6.14$) andα(β=−27.73,t(45)=−3.47,p=0.001,$BF_{\text{inclusion}}=16.65$) recorded during eyes-open rest are reliably different from zero. These findings suggest that higher mid-frontal theta oscillations and lower mid-frontal alpha oscillations recorded during eyes-open rest predict trait anxiety, as measured with behavioral inhibition scale.

**Table 1.** Regression model for mid-frontal (Fz) and mid-parietal (Pz) resting-state neural oscillations (left) and power (right) predicting BIS scores.

Fz	Oscillations			Power		
Predictors	beta	t	$BF_{inclusion}$	beta	t	$BF_{inclusion}$
θ Eyes Open	0.33	$2.51^{*}$	6.14	0.93	$2.45^{*}$	3.6
θ Eyes Closed	-0.05	-0.32	1.13	-0.52	-1.21	1.36
α Eyes Open	-0.46	$\text{-}3.47^{*}$	16.65	-0.94	$\text{-}3.25^{*}$	7.07
α Eyes Closed	0.3	1.82 †	3.22	0.49	1.97 †	2.32

Pz	Oscillations			Power		
Predictors	beta	t	$BF_{inclusion}$	beta	t	$BF_{inclusion}$
θ Eyes Open	0.02	0.09	0.6	0.43	0.66 †	0.91
θ Eyes Closed	0.14	0.79	0.81	0.25	0.39	1.08
α Eyes Open	-0.4	$\text{-}2.23^{*}$	2.44	-1.04	$\text{-}2.59^{*}$	2.75
α Eyes Closed	0.19	1.13	0.94	0.45	1.44	1.54

Each predictor for oscillation analysis is the proportion of oscillations ($P_{episode}$) of band-average neural activity, and each predictor in power analysis is the power of a band-average neural activity recorded at rest during eyes-open or eyes-closed conditions, respectively.*p<0.05;†p<0.1. Overall, the Fz oscillation model explained 26% of the variance (p=0.003), the Fz power model explained 21% of the variance (p=0.008), the Pz oscillation model explained 12% of the variance (p=0.055), and the Pz power model explained 14% of the variance (p=0.013).

Multiple regression for BIS and resting-state power model is also summarized inTable 1. Similar to the multiple regression with oscillation measures, the model with all four power predictors was also significant, with adjusted$R^{2}=0.21$,$R^{2}=0.28$,F(4, 41)=3.96,p=0.008. The main predictors are the same in the power model as we found in the oscillation model. More precisely, theθ(β=17.26,t(45)=2.45,p=0.02,$BF_{\text{inclusion}}=3.6$) andα(β=−15.26,t(45)=−3.25,p=0.002,$BF_{\text{inclusion}}=7.07$) recorded during eyes-open rest are reliably different from zero, also supported by Bayesian statistics. The fact that BOSC and power measures both predict the BIS scores with a similar pattern strengthens the claim that resting-state neural activity holds valuable information for anxiety processes. Moreover, BOSC measures suggest that there is legitimate rhythmicity underlying the effect.

### 3.2 BIS and resting-state neural oscillation and power models at Pz

Multiple regression for BIS and Pz resting-state model is summarized inTable 1. The model with four oscillatory predictors (PzθEyes Open,θEyes Closed,αEyes Open,αEyes Closed) fell just short of significance, with adjusted$R^{2}=0.12$,$R^{2}=0.198$,F(4, 41)=2.53, andp=0.055. The only significant predictor from the Pz oscillation model was alpha recorded during eyes-open rest (β=−0.4,t(45)=−2.23,p=0.032,$BF_{\text{inclusion}}=2.44$). Contrary to the Pz oscillation model, the similar model with all four power predictors was significant with adjusted$R^{2}=0.14$,$R^{2}=0.22$,F(4, 41)=2.82, andp=0.037. As in the oscillation model, the only significant predictor in the power model was alpha recorded during eyes-open rest (β=−1.04,t(45)=−2.59,p=0.013,$BF_{\text{inclusion}}=2.75$). However, the Bayes Factors suggest there is not quite enough evidence for eyes-open alpha oscillation ($BF_{\text{inclusion}}=2.44$) or power ($BF_{\text{inclusion}}=2.75$) recorded at the Pz electrode to be included in the model, potentially suggesting that we may be recording more of classic “resting visual alpha” at Pz, leaving the frontal midline neural activity exclusively important in predicting anxiety.

### 3.3 BIS and resting-state neural oscillation and power models at Fz and Pz combined

Having observed that both Fz and Pz electrodes shared the significant predictors, we ran a post hoc, combined regression with all eight predictors (Fz:θEyes Open,θEyes Closed,αEyes Open,αEyes Closed, and Pz:θEyes Open,θEyes Closed,αEyes Open,αEyes Closed), summarized inTable 2. The model was significant, with adjusted$R^{2}=0.23$,$R^{2}=0.37$,F(8, 37)=2.698, andp=0.019. Interestingly, the only significant predictor in this combined analysis proved to be theta oscillations recorded at Fz electrode during eyes-open rest (β=0.39,t(45)=2.53,p=0.016,$BF_{\text{inclusion}}=3.26$).

**Table 2.** The combined regression model for mid-frontal (Fz) and mid-parietal (Pz) resting-state neural oscillations (left) and power (right) predicting BIS scores.

		Oscillation			Power		
Predictors		beta	t	$BF_{inclusion}$	beta	t	$BF_{inclusion}$
Fz	θ Eyes Open	0.39	$2.53^{*}$	3.26	1.07	$2.27^{*}$	2.43
	θ Eyes Closed	-0.2	-0.88	0.69	-1.41	$-2.36^{*}$	1.86
	α Eyes Open	-0.45	-1.31	2.03	-0.54	-1.0	2.16
	α Eyes Closed	0.42	1.34	1.17	0.68	1.41	1.5
Pz	θ Eyes Open	-0.26	-1.13	0.87	-0.58	-0.75	1.11
	θ Eyes Closed	0.3	1.42	0.92	1.7	2.08 †	1.92
	α Eyes Open	0.07	0.19	1.11	-0.57	-0.8	1.81
	α Eyes Closed	-0.22	-0.73	0.78	-0.33	-0.54	1.3

Each predictor for oscillation analysis is the proportion of oscillations ($P_{episode}$) for band-average neural activity, and each predictor in power analysis is the power of a band-average neural activity recorded at rest during eyes-open or eyes-closed conditions, respectively.*p<0.05;†p<0.1. Overall, the Fz oscillation model explained 26% of the variance (p=0.003), the Fz power model explained 21% of the variance (p=0.008), the Pz oscillation model explained 12% of the variance (p=0.055), and the Pz power model explained 14% of the variance (p=0.013).

For power, similarly, the combined Fz and Pz regression model with eight predictors was significant, with adjusted$R^{2}=0.248$,$R^{2}=0.38$,F(8, 37)=2.86, andp=0.014. Interestingly, in the combined power model, the prediction was driven by two significant predictors (in contrast to the one significant predictor in the oscillation model), which are theta oscillations recorded at Fz during eyes-open rest (β=1.07,t(45)=2.27,p=0.03,$BF_{\text{inclusion}}=2.43$) and during eyes-closed rest (β=−1.41,t(45)=−2.36,p=0.02,$BF_{\text{inclusion}}=1.86$). Surprisingly, neither of these predictors were conclusive with Bayesian analysis.

This approach allowed us to see whether each region’s activity remained significant when controlling for the other. Our analysis showed that although parietal alpha power predicted BIS scores in isolation, it did not remain significant when frontal measures were included. This suggests that the relationship of the parietal alpha with BIS may be weaker or more context dependent relative to frontal midline activity. This distinction clarifies the dominant role of frontal midline rhythms in anxiety-related inhibition.

### 3.4 Reliability analysis

To address concerns regarding the reliability of the findings based on the 2-minute resting-state EEG epochs for each condition, we conducted a robustness check using bootstrapping with sub-sampled data segments ranging from 10% to 100% of the total recording duration. For each percentage of data used, we performed 100 bootstrapped linear regressions to predict BIS scores using mid-frontal (Fz) and mid-parietal (Pz) theta and alpha oscillations recorded during eyes-open (EO) and eyes-closed (EC) conditions.

As shown inFigure 3, the average beta values plateaued as the percentage of data increased. The results indicate that using between 30% and 50% of the total EEG recording duration provides reliable and stable predictions of trait anxiety. Beyond this range, increasing the data duration did not significantly improve the predictive power of the models.

**Fig. 3.**
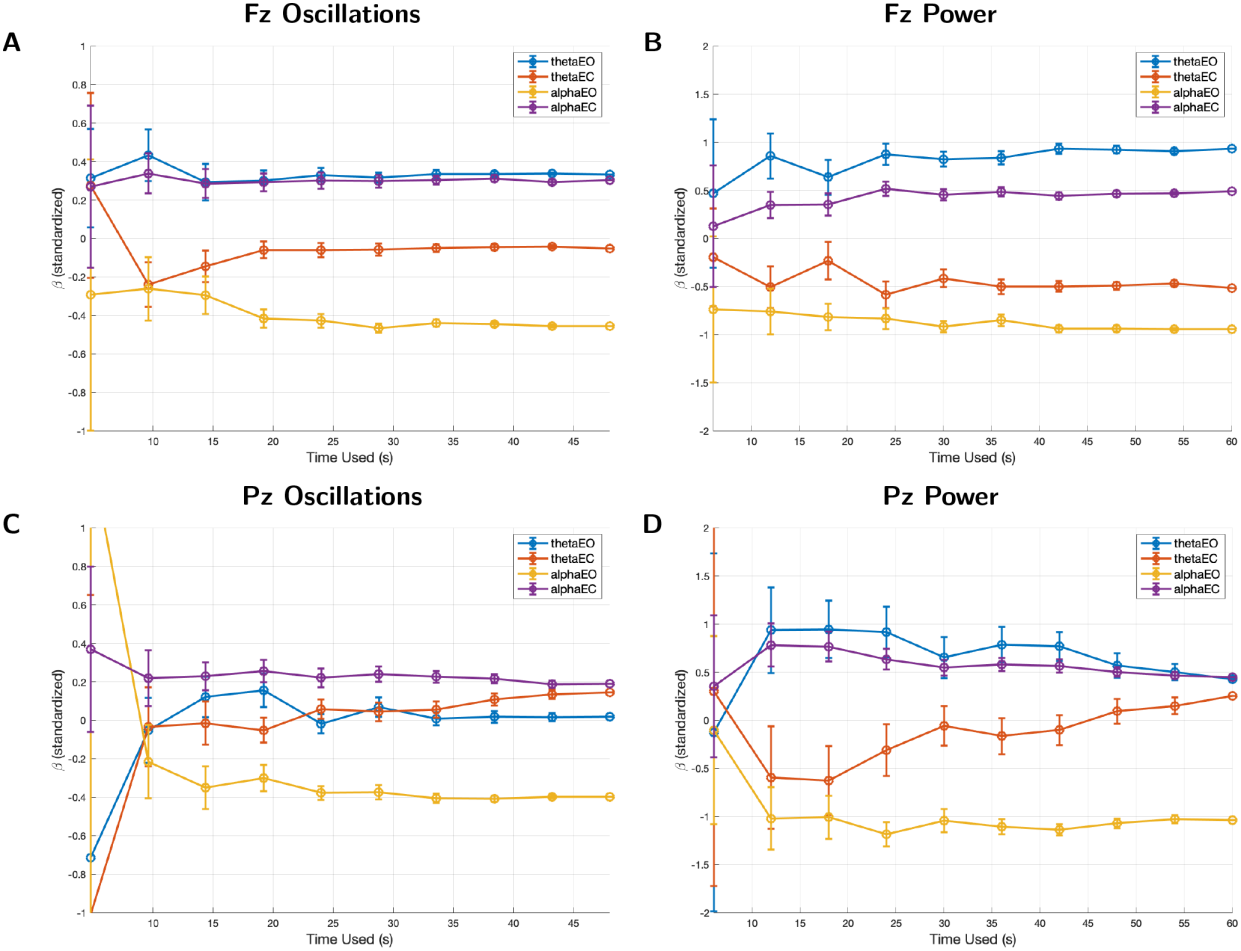
Reliability analysis was performed using bootstrapping with sub-sampled EEG data segments ranging from 10% to 100% of the total recording duration (48 seconds for oscillation and 60 seconds for power analysis). For each percentage window of data used, we performed 100 bootstrapped linear regressions to predict BIS scores using mid-frontal (Fz) and mid-parietal (Pz) theta and alpha oscillations (A and C) and power (B and D) recorded during eyes-open (EO) and eyes-closed (EC) conditions. The error bars represent the 95% confidence intervals of the standardized beta coefficients.

These findings suggest that the original 1-minute (total of 2-minute per condition) EEG epochs used in the main analysis are sufficient to produce robust results. The bootstrapping approach further supports the stability of the findings, reducing the likelihood that the observed relationships between mid-frontal oscillations and BIS scores are due to chance or variability in the data.

### 3.5 Correlations between eyes-open resting-state oscillations and anxiety questionnaires

**Fig. 4.**
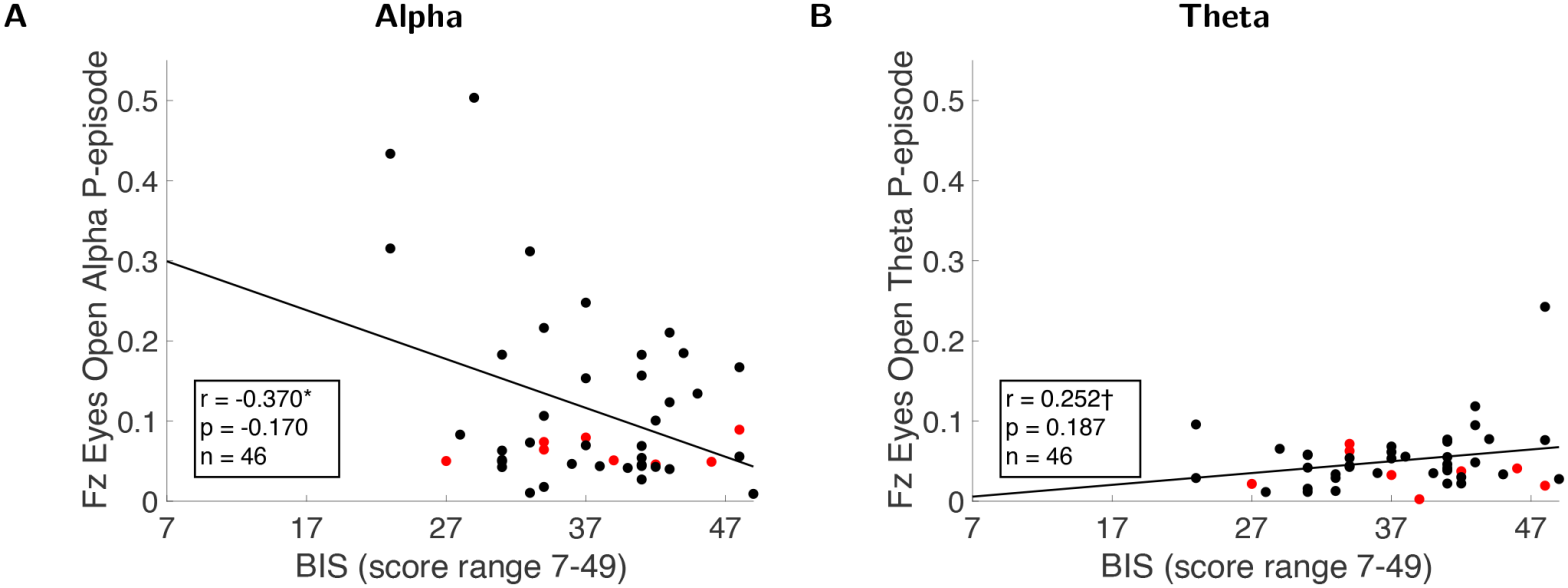
Scatter plots for Pearson correlations (df=45) between eyes-open alpha (A) and theta (B) oscillations recorded at Fz electrode and BIS scores. Red dots represent individuals with self-reported anxiety diagnosis.

Although BIS scores are not reliably predicted by oscillations recorded at the Pz electrode, the negative correlations of all questionnaire scores with eyes-open resting states, both for frontal (Fz) (Fig. 4A) and parietal (Pz) electrodes, are significant in theαrange (Table 3). Interestingly,θoscillations are not correlated with anxiety questionnaires; only a marginal but non-significant correlation is found between frontal midlineθrecorded during eyes-open rest and BIS scores (p=0.091) (Fig. 4B).

**Table 3.** Pearson correlations between alpha and theta oscillations recorded at Fz (left) and Pz (right) electrodes during eyes-open and eyes-closed rest, and BIS, STAI Trait, and TIPI neuroticism scores.

	Fz					Pz			
	*df*	α EO	α EC	θ EO	θ EC	α EO	α EC	θ EO	θ EC
BIS	45	$\text{-}0.37^{*}$	0.25 †	0.25 †	0.12	$\text{-}0.34^{*}$	0.22	-0.17	0.17
STAI Trait	50	$\text{-}0.31^{*}$	-0.002	0.03	0.05	$\text{-}0.28^{*}$	0.15	-0.15	0.06
TIPI	50	$\text{-}0.36^{*}$	0.02	-0.02	0.05	-0.25 †	0.16	-0.16	0.15

*p<0.05,†p<0.1.

**Fig. 5.**
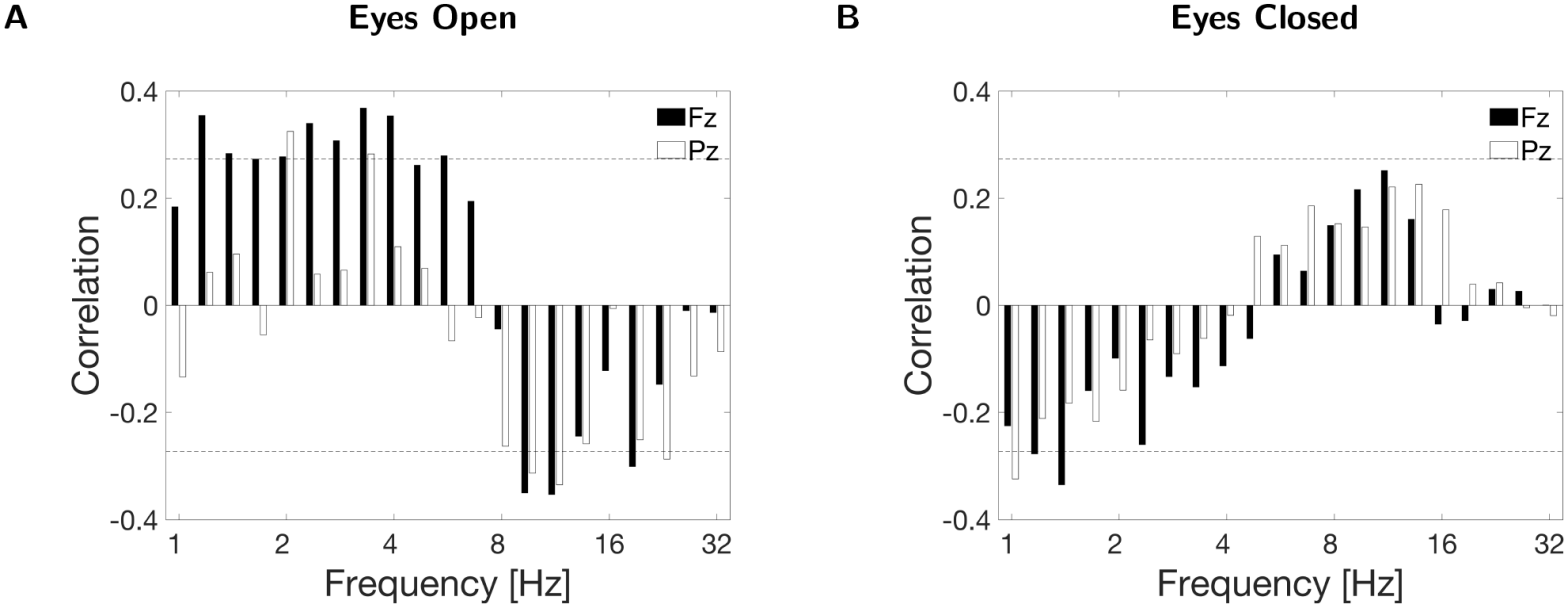
Pearson correlation (df=45) for BIS anxiety scores correlating with resting-state neural oscillations ($P_{episode}$) recorded at Fz and Pz electrodes during eyes-open (A) and eyes-closed (B) conditions plotted for all frequencies. The dashed lines denote the significance thresholds (p<0.05).

We also plotted correlation values as functions of all frequencies at electrodes Fz and Pz (Fig. 5). Generally, the correlation results at all frequencies analyzed confirmed the results of the frequency band-specific (band-average) analyses. However, it also becomes evident that the effects are not limited to theta and alpha ranges. Frequencies in the range of 1–8 Hz (which includes theta band) are positively associated with BIS, whereas frequencies in the range of 8–32 Hz (which includes alpha band) are more negatively associated with the BIS scores. This type of analysis highlights a pitfall of arbitrarily defining frequency ranges for rhythms such as theta and alpha. For example, although theta is traditionally considered to span 4–8 Hz, the correlation at all sampled frequencies demonstrated inFigure 5Areveals that the 8 Hz frequency aligns more closely with the negative correlation trend of the “alpha peak” rather than a positive correlation pattern of the “theta peak.” Furthermore, the marginal but non-significant nature of the theta-band average correlation with BIS scores (p=0.09) suggests that removing 8 Hz from the theta range could potentially reveal an underlying significant correlation with more frequency specificity. Even though we refrain from making such adjustments post hoc, this is an important consideration for future research.

### 3.6 Alpha asymmetry and anxiety

The multiple regression for BIS and resting-state alpha oscillatory asymmetry model is summarized inTable 4and also visualized inFigure 6. The model with all four predictors (F4–F3αasymmetry EO, F4–F3αasymmetry EC, F8–F7αasymmetry EO, and F8–F7αasymmetry EC) was not significant (p=0.107), nor were any of the individual predictors. The same analysis on power instead of$P_{episode}$(Table 4) was also not significant (p=0.565), nor were any of the particular predictors. These findings suggest that frontal alpha asymmetry is not a reliable predictor of BIS scores.

**Fig. 6.**
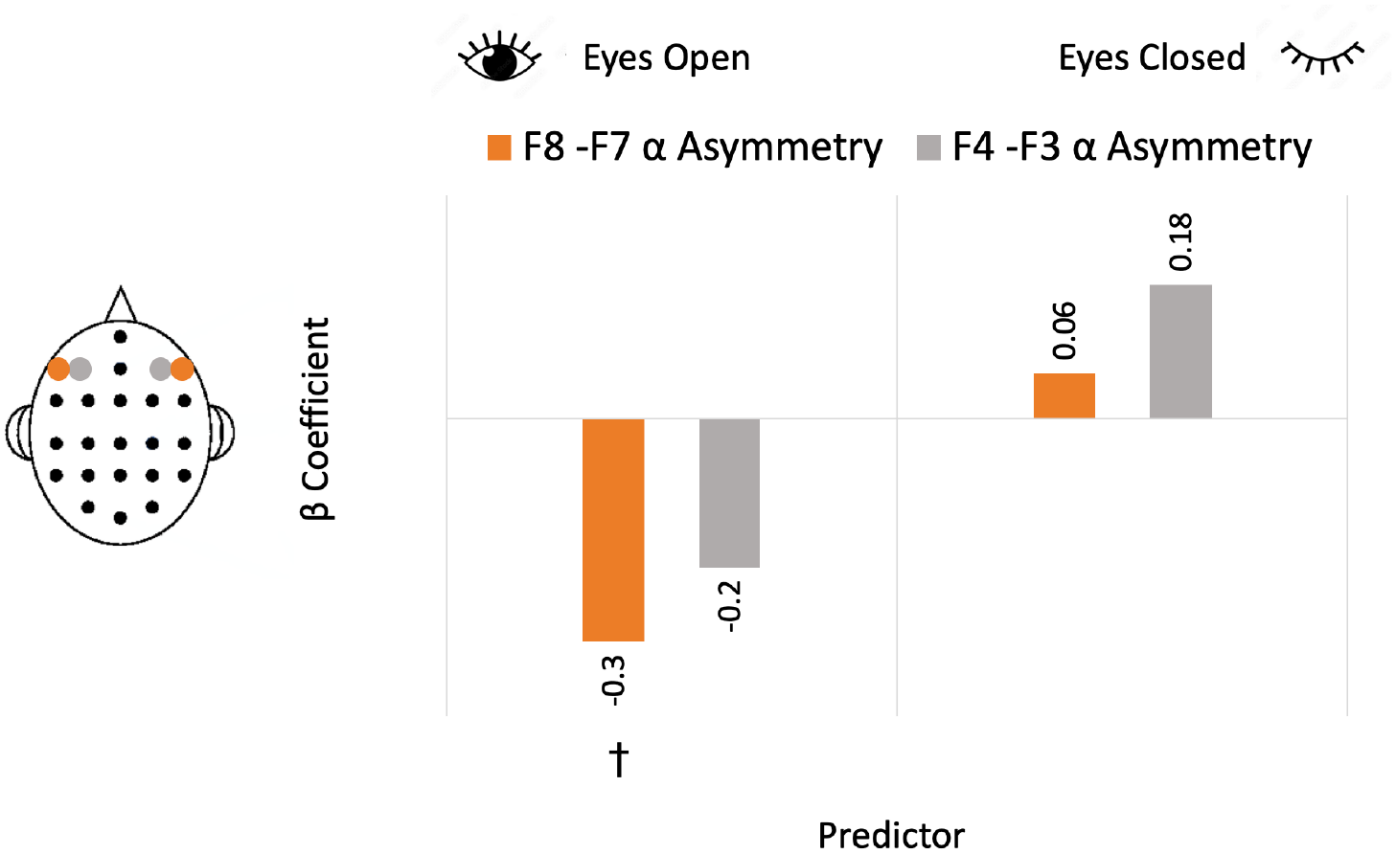
The representation of the multiple regression model predicting BIS scores with lateral-frontal (F8–F7) and mid-frontal (F4–F3) alpha oscillatory ($P_{episode}$) asymmetry measured during eyes-open and eyes-closed resting states. Orange bars represent beta coefficients for lateral-frontal (F8–F7) alpha oscillatory asymmetry measures predicting BIS, and gray bars represent beta coefficients of mid-frontal (F4–F3) alpha oscillatory asymmetry measures predicting BIS scores.

**Table 4.** The regression model for mid-frontal (F4–F3) and lateral-frontal (F8–F7) resting-state neural oscillatory activity (left) and power asymmetry (right) predicting BIS scores.

	Oscillations			Power		
Predictors	beta	t	$BF_{inclusion}$	beta	t	$BF_{inclusion}$
F4–F3 α asym EO	-0.2	-1.14	0.59	-0.61	-1.28	0.3
F4–F3 α asym EC	0.18	1.0	0.55	0.56	1.16	0.29
F8–F7 α asym EO	-0.3	-1.77 †	1.66	-0.02	-0.04	0.24
F8–F7 α asym EC	0.06	0.35	0.45	0.08	0.15	0.26

Each predictor represents the band-average neural activity ($P_{episode}$or power) for the left electrode (F7 or F3) subtracted from the right electrode (F8 or F4), recorded during eyes-open or eyes-closed resting states.*p<0.05;†p<0.1.

Following theCoan and Allen (2003)logic about BIS system complexity, as an exploratory step, we also looked into different BIS subscales split according to the model suggested byJohnson et al. (2003). To recap, it was suggested that if more right hemisphere alpha activity is triggered by the BIS threat-related subsystem, and not by anxiety-related processes, the relationship between full BIS scale and right frontal alpha activity would be sample- and situation-dependentCoan and Allen (2003).

To directly test this idea, we correlated scores from different BIS subscales with our mid-frontal (F4–F3) and lateral-frontal (F8–F7)αasymmetry measures. We found greater lateral-frontal (F8–F7) oscillatoryαasymmetry during eyes-open rest to be negatively correlated with overall BIS scores (R=−0.35,p=0.02). However, after splitting the BIS scale, this correlation only stayed significant for the*J-FFFS*BIS subscales (R=−0.4,p=0.01), but not for the*J-BIS*one (R=−0.24,p=0.11). This dissociation suggests that individuals with more activated fear-related processes, but not exclusively anxiety-related processes, had lower rightward alpha asymmetry during eyes-open rest (Table 5). The relationship was not found between power asymmetry measures and BIS scales.

**Table 5.** Pearson correlation between alpha oscillatory ($P_{episode}$) asymmetry recorded at lateral-frontal (F8–F7) & mid-frontal (F4–F3) electrode pairs during eyes-open and eyes-closed rest with BIS scale and subscale scores.

	Oscillations	R	p
BIS	F7–F8 α Asym EO	−0.352 ^*^	0.016
	F7–F8 α Asym EC	−0.032	0.834
	F4–F3 α Asym EO	−0.203	0.176
	F4–F3 α Asym EC	0.122	0.418
J_BIS	F7–F8 α Asym EO	−0.238	0.111
	F7–F8 α Asym EC	0.048	0.754
	F4–F3 α Asym EO	−0.121	0.424
	F4–F3 α Asym EC	0.134	0.376

J_FFFS	F7–F8 α Asym EO	−0.396 ^*^	0.006
	F7–F8 α Asym EC	−0.158	0.295
	F4–F3 α Asym EO	−0.258	0.084
	F4–F3 α Asym EC	0.048	0.753

* Denotesp<0.05.

## 4 Discussion

Taking an individual differences approach, we tested whether neural activity recorded at the single frontal midline (Fz) and parietal midline (Pz) electrodes would predict behavioral inhibition as a measure of trait anxiety. We found that higher BIS scores were associated with increased frontal midline theta and decreased frontal midline alpha oscillations during eyes-open rest. Additionally, resting-state parietal midline alpha power, but not oscillations, explained less variance in BIS scores (with inconclusive Bayes factors) than the frontal midline theta and alpha power and oscillations. Further, we discovered that frontal alpha asymmetry (power or oscillations) did not predict BIS scores. Notably, correlation analysis showed that frontal midline oscillatory alpha asymmetry during eyes-open rest was negatively correlated with the fear-related BIS subscale (*J-FFFS*), and not with the anxiety-related subscale (*J-BIS*).

### 4.1 Frontal midline theta and anxiety

Associating higher frontal midline theta with increased trait anxiety and the behavioral inhibition system aligns with Gray and McNaughton’s septohippocampal theory (Gray, 1982;Gray & McNaughton, 2000), extending its application to resting-state EEG activity. Earlier task-related studies found that human hippocampal “theta” frequency at right frontal electrodes (F8) in the range of 4–12 Hz (named goal-conflict-specific EEG rhythmicity (GCSR)) was positively correlated with neuroticism and trait anxiety and was reduced by all key anxiolytic drugs (Neo et al., 2011;Shadli et al., 2020,^2021^).Shadli et al. (2021)also found that goal-conflict-related “theta” rhythm was higher in individuals with clinical anxiety levels. However, despite its theoretical significance, the terminology of referring to GCSR as “theta” rhythm warrants some consideration, given that its range (4–12 Hz) extends beyond the traditionally defined human theta band (4–8 Hz).

In the more classical view of human theta rhythms, meta-analysis byCavanagh and Shackman (2015)concluded that frontal midline (Fz) theta (4–8 Hz) oscillations reflect increased cognitive control and relate to greater trait anxiety and avoidant behavior during goal-conflict arousing tasks. However, they could not find any clinical significance of this neural marker. In contrast,Cavanagh et al. (2017)found that error- and conflict-related neural oscillations in the theta band can reliably differentiate individuals with generalized anxiety disorder from healthy controls.

BIS theory also suggests that unresolved goal conflict subjectively manifests as anxious rumination (Corr, 2008;Gray & McNaughton, 2000).Andersen et al. (2009)experimentally linked this idea to theta activity, showing that personally meaningful goal conflict, potentially triggering anxious rumination, indeed enhanced the scalp-wide EEG theta (4–6 and 6–8 Hz) power and coherence, compared with less personally meaningful, nominal ruminations. Surprisingly, this increase was not associated with participants’ BIS levels. On the other hand,Moore et al. (2006)found that scalp-wide theta power and coherence effectively distinguished between high and low BIS participants. However, in this study, goal conflict only increased theta coherence across various regions, whereas theta power showed a non-significant trend, suggesting that the differentiation in BIS levels was primarily driven by theta coherence measures, and not power. More interestingly, despite the hypothesized frontal midline theta activation, neither study observed region-specific increases in theta power; significant results only emerged when scalp-wide averages were analyzed.

These studies collectively suggest that the septohippocampal system is activated during goal-conflict tasks in humans. Extending this perspective, our findings suggest that in individuals with high trait anxiety, this system may be more generally activated. It appears to remain in a heightened “ready” state to inhibit behaviors fraught with potential negative outcomes, even during rest. Additionally, it is arguably more plausible to detect periods of internal anxious rumination when individuals are resting rather than engaging in goal-conflict tasks. We speculate that individuals with higher BIS may be more prone to experiencing internalized anxious rumination during rest, and that increased FMT could reflect such intrusive thoughts and internalized scenarios of goal conflict. From this perspective, our finding of an association between resting-state theta rhythm and BIS scores might indicate a stronger link to these cognitive processes compared with the null correlation between task-induced anxious rumination and BIS reported byAndersen et al. (2009).

Our findings seem at odds withCaplan et al. (2015), who used the same BOSC method but found virtually no evidence of theta oscillations during rest. So, what accounts for the robust effects in the theta band at rest in our study? A key difference between the two studies lies in the duration of the resting-state recordings. In theCaplan et al. (2015)study, each eyes-open or eyes-closed segment lasted only 5 seconds. In contrast, we used much longer 1-minute segments for each condition. This extended duration may have given us a better opportunity to capture the kinds of internalized cognitive and emotional processes, such as anxiety-related rumination, that we associate with FMT activity. This aligns with the idea that when people ruminate or dwell on anxious thoughts, those thoughts typically unfold over a longer time frame. It is unlikely that such internalized processes would arise and stabilize within just 5 seconds. By using longer recording segments, we may have provided the necessary conditions for these ruminative thoughts and the associated theta activity to emerge. If frontal theta reflects prolonged internal processes such as rumination, then short recordings simply would not allow enough time for this signal to manifest. Taken together, our findings suggest that longer resting-state recordings may be essential to accurately detect theta activity related to internalized thought processes such as rumination.

Our results also differ from older studies that reported no frontal midline theta activity during rest and lower FMT in more anxious individuals performing mental arithmetic tasks (Mizuki et al., 1984;Suetsugi et al., 2000). As mentioned in the introduction, some of these earlier studies relied on EEG recordings captured on paper and analog tape recorders, which likely limited the detection of finer neural patterns, prompting us to interpret their findings carefully. Moreover, in these studies, anxiety was not measured with behavioral inhibition scales but with the Maudsley Personality Inventory and STAI. To our knowledge, this is the first paper analyzing resting frontal midline theta with behavioral inhibition scales and methods that are selective for not just spectral power but also rhythmicity.

***Clinical Relevance.***In a comprehensive review,McLoughlin et al. (2022)found that FMT is consistently elevated in individuals with certain psychiatric conditions, including generalized anxiety disorder (GAD), but primarily during tasks involving uncertainty or cognitive conflict rather than rest. By contrast,Kopanska et al. (2024), reviewing both resting and task-based EEG rhythms in neuropsychiatric disorders, found no consistent evidence linking theta oscillations with anxiety disorders.

Direct clinical research examining anxiety-related FMT during resting-state EEG remains limited. One notable exception isXing et al. (2016)who found increased frontal midline theta coherence in individuals with generalized social anxiety disorder during rest, a pattern that aligns with our findings. Theta rhythms have also been linked to other mental health disorders, including depression, a common comorbidity of anxiety disorders.Gold et al. (2013)examined FMT and frontal alpha asymmetry in a clinically depressed sample and found that although reliability was adequate, FMT was only weakly correlated with anxiety measures.Zhang et al. (2022)also explored whether resting theta rhythm could serve as a useful biomarker for distinguishing between anxiety and depressive disorders, and found that theta power (4–7 Hz) was significantly stronger in individuals with depression than in those with anxiety, though this effect was limited to certain electrodes (e.g., F3, O2, T3, P3, P4, FP1, FP2, and F8). Frontal theta activity has additionally been explored as a therapeutic target. For example, a study byLi et al. (2022)demonstrated that continuous theta-burst stimulation (cTBS) over the right dorsolateral prefrontal cortex improved anxiety symptoms in patients with generalized anxiety disorder, accompanied by increases in alpha oscillations.

Collectively, these findings suggest that although theta activity may have transdiagnostic relevance, its relationship with clinical anxiety remains debated and may depend on factors such as experimental conditions, diagnostic category, and electrode site. Notably, most clinical studies rely on anxiety symptom scales, which differ substantially from the behavioral inhibition scale used here, as it targets a specific anxiety-related process (Gray & McNaughton, 2000;Shadli et al., 2021). Our findings complement this body of work by demonstrating that FMT is also elevated during eyes-open resting-state EEG in individuals with high trait anxiety, a known risk factor for developing psychiatric disorders. This supports the idea that FMT may serve not only as a clinical biomarker but also as a potential vulnerability marker, thereby extending the relevance of clinical observations to a resting, task-free context and highlighting its potential utility in early identification of individuals at risk for anxiety-related disorders.

### 4.2 Frontal midline alpha and anxiety

Measuring frontal midline alpha during rest is not common in EEG research. As far as we are aware, this is the first report of frontal midline alpha being investigated and associated with the behavioral inhibition system. Due to the higher popularity of the approach-withdrawal motivational model and its association with frontal alpha asymmetry, while there has been a large amount of research done in the alpha activity and BIS domain, researchers almost always use asymmetry coefficients in their analysis and disregard the single-electrode information. However, our findings clearly suggest that alpha at midline electrodes also convey valuable information and explain a substantial part of the variance in BIS scores. As mentioned in the introduction, there might be good reasons why earlier studies favored asymmetry measures over single-electrode ones. When subtracting right hemisphere neural activity from the left, the power measurement is calibrated, and some of the intrinsic variability of the EEG signal is corrected. However, analysis methods such as BOSC, which are calibrated using the signal’s own background power spectrum, make single-electrode analyses more feasible and comparable, and potentially more accurate than the right–left asymmetry measure.

Consider, now, the alpha “idling” hypothesis. Just as greater occipital alpha is robustly associated with the inhibition of task-irrelevant visual stimuli (Foxe & Snyder, 2011), it is plausible that greater frontal midline alpha reflects the inhibition of processes governed by the frontal brain regions, such as rumination, anxiety, cognitive control, and emotion regulation. Consequently, the observation that lower frontal midline alpha predicts behavioral inhibition or trait anxiety could be attributed to increased cortical activation in anterior brain regions involved in inhibition and cognitive control, such as prefrontal cortex and anterior cingulate cortex (Bush et al., 2000;Diamond, 2013). Further, following the assumption that alpha power is an inverse measure of cortical activation (Davidson, 1992), lower alpha power in these regions suggests their greater activation. However, these explanations should be interpreted with caution due to the limited spatial resolution of EEG and the aforementioned arguments against the idea that alpha activity is always an inverse of cortical activation.

Although posterior alpha activity has long been associated with arousal and alertness (Klimesch et al., 2007), our findings indicate that only parietal-recorded eyes-open alpha power predicted BIS scores, with no significant relationship found for oscillations. Furthermore, Bayesian analyses did not support the alpha power and BIS results, suggesting that the evidence for such a relationship is weak and insufficient to make a confident claim. Moreover, in a post hoc analysis combining Fz and Pz electrode predictors in one regression model, only frontal midline theta (both power and oscillation) emerged as the primary predictor of BIS, out of all eight predictors. Bayesian analysis further confirmed that frontal midline theta oscillations were the only well-supported predictor.

Given the statistically weak results for the parietal electrode power measures, it is possible that the Pz eyes-open alpha recorded in our study reflects classic visual alpha activity (Klimesch, 1999), which we speculate is less closely tied to anxiety-related neural mechanisms. Although speculative, it is also possible that rhythmicity differentiates the functional roles of frontal and posterior alpha in relation to anxiety. Anxiety-related frontal alpha and theta may be driven by rhythmic processes, whereas posterior alpha could reflect non-rhythmic power, contributing to the variability in results across studies. This distinction could help explain why global alpha measures, which collapse both electrodes and signal types, have produced inconsistent findings in the literature. Separating these components, as done with the BOSC method, offers a more nuanced understanding and could clarify the distinct contributions of oscillatory and non-oscillatory activity to anxiety-related neural dynamics. Future studies could further test this by specifically isolating rhythmic components to better understand their distinct role in anxiety-related neural activity.

### 4.3 Frontal alpha asymmetry and anxiety

Evidence also suggests that the right inferior frontal cortex is important in behavioral inhibition (Aron et al., 2014). The assumption that alpha power inversely reflects cortical activation would imply that reduced alpha oscillations at the right frontal electrodes would indicate heightened activity in this area. Yet, our frontal alpha asymmetry analysis did not support this line of reasoning. We examined frontal alpha asymmetry at two electrode pairs but found that asymmetry at neither location sufficiently explained individual variability in BIS scores. These null results align with the conclusions of a recent systematic review byFirth et al. (2024), who found that among the reviewed studies, most reported no consistent relationship between resting frontal alpha asymmetry and behavioral inhibition. This convergence of evidence suggests that frontal alpha asymmetry may not be a reliable neural correlate of BIS sensitivity in resting-state EEG.

Although the overall regression was not significant, there was a weak trend suggesting that lateral-frontal (F8–F7) alpha asymmetry during eyes-open rest might predict BIS scores. However, an exploratory correlational analysis, which involved dividing the BIS scale into two subscales, revealed that only the FFFS-related subscales was significantly associated with lateral-frontal (F8–F7) asymmetry. This finding may help explain the mixed results often reported in the literature. The updated septohippocampal theory of anxiety distinguishes between two sub-processes within the avoidance/withdrawal dimension (previously grouped under BIS). Since then, some researchers have suggested that the BIS scale does not align well with this updated theory and should be revised to clearly separate FFFS (fear-related) processes from BIS (anxiety-related) processes (Heym et al., 2008;Johnson et al., 2003). Given that earlier studies did not consider such a distinction, the inconsistency in findings could reflect the differential representation of these two constructs, with alpha asymmetry measures being differently associated with FFFS and BIS. This was evident in our exploratory analysis, where only the FFFS subscale, but not the BIS subscale, significantly correlated with alpha asymmetry recorded at lateral-frontal (F8–F7) electrodes.

Another possibility is that previous studies, using conventional power measures, may not have captured the rhythmic subset of activity showing frontal alpha asymmetry. If only non-rhythmic alpha asymmetry is associated with BIS or FFFS, but the rhythmic activity is not, these studies may have detected the true effect only some of the time, contributing to the mixed results. This may be addressed in the future by using both BOSC (or similar methods) and conventional power measures when studying frontal alpha asymmetry, as we did here. Although neither power- nor oscillatory-asymmetry reliably predicted BIS scores in our study, comparing these methods in future resting-state EEG analyses could provide additional clarity on the true nature of the effect.

### 4.4 Resting state: eyes-open versus eyes-closed rest

Our findings also reveal significant differences in resting-state power and oscillatory activity between eyes-open and eyes-closed conditions, reinforcing the idea that these two states are functionally distinct and should not be conflated in analyses. Although alpha-rhythm researchers sinceBerger (1929)have known that at least posterior alpha activity changes drastically when the participant opens or closes their eyes, many studies of resting-state activity still overlook these distinctions, sometimes even combining analysis of both states or using one as a baseline for the other. For instance,Knyazev et al. (2003)reported an opposite pattern, where relative alpha power was positively associated with behavioral inhibition (i.e., trait anxiety) as measured by the Gray–Wilson Personality Questionnaire and Eysenck Personality Inventory in mostly females. However, their analysis aggregated alpha power across both eyes-closed and eyes-open states and six electrode locations (global alpha). Similarly,Knyazev et al. (2004)found the same association in males, but only used eyes-closed rest to measure the “resting state.” Moreover, in further analysis, they used activity during eyes-closed rest as a baseline to then evaluate the activity of the eyes-open resting states (before beep, after beep, or after word presentation). Given the drastic differences in eyes-open and closed resting-state oscillatory patterns, such analysis might yield confounded results or overlook a nuanced relationship between resting states (eyes open and eyes closed), frontal and parietal neural activity, and trait anxiety.

The greater predictive power of eyes-open resting-state oscillations for anxiety is consistent with the idea that eyes-open rest may be a more arousing state for individuals with high trait anxiety, whereas eyes-closed rest may be more relaxing. If FMT serves as a biomarker of behavioral inhibition, individuals with high trait anxiety may exhibit increased FMT during eyes-open rest as they prepare to inhibit unwanted stimuli or behaviors. In eyes-closed rest, when the inhibitory system is more at ease, FMT activity may decrease. In line with this reasoning, future research on resting state and individual differences should examine both eyes-open and eyes-closed resting conditions to capture these variations.

Overall, our findings suggest that anxiety-related lower frequency power and oscillations are detectable during more vigilant and potentially meaningful resting states, such as eyes open, especially in individuals with high-trait anxiety. Although both frontal midline alpha and frontal midline theta oscillations were found to be reliable predictors of behavioral inhibition, our results show that frontal midline theta is statistically more robust. This strengthens the case for frontal midline theta oscillations, which are strongly supported by theoretical frameworks, as a reliable neural correlate of behavioral inhibition (Gray & McNaughton, 2000;Shadli et al., 2021).

## 5 Limitations and Broader Downstream Implications

A potential limitation of the current study is that self-reports of trait anxiety can be influenced by biases and measurement errors (Kreitchmann et al., 2019;Wall & Lee, 2022). Future research could benefit from integrating alternative measures of anxiety, such as heart rate variability (Chalmers et al., 2014) or cortisol (Zorn et al., 2017) to mitigate these limitations. However, it is important to note that these measures have their own limitations and are primarily biomarkers of state rather than trait anxiety (Bennett, 2022). This underscores the need for more robust and reliable measures of both trait and state anxiety, and we hope that our findings contribute to advancing the development of such measures in the field.

It is also important to clarify that when we discuss frontal midline alpha and theta oscillations predicting anxiety, we refer to anxiety as a psychological trait, not a clinical disorder. Our study measured trait anxiety in healthy individuals, and we do not claim that these oscillatory activities are linked to any specific anxiety psychopathologies.

Given that trait anxiety is a known risk factor for anxiety disorders, future studies could examine the link between specific anxiety diagnoses and frontal midline alpha and theta oscillations. This could help develop objective biomarkers to improve diagnostic accuracy, which remains a challenge in psychiatry, where diagnoses still rely heavily on subjective assessments. However, exploring this in clinical settings goes beyond the scope of the current study.

## 6 Conclusion

In conclusion, this study demonstrates that both frontal midline theta and frontal midline alpha oscillations play a significant role in predicting trait anxiety and behavioral inhibition, particularly during eyes-open rest, where individuals with high trait anxiety may experience heightened arousal. Supported by stronger statistical results and robust theoretical evidence, the findings linking frontal midline theta to the behavioral inhibition system provide meaningful insights into the neural mechanisms underlying anxiety. The results also highlight the importance of distinguishing between eyes-open and eyes-closed resting states in future studies. Additionally, the use of the BOSC method to separate rhythmic from non-rhythmic activity provided clearer insights into the neural dynamics linked to anxiety, emphasizing the need to account for both signal types when studying traits such as anxiety. Together, these findings provide valuable insights into the underlying neural mechanisms of anxiety and behavioral inhibition and highlight key considerations for future research in this area.

## Data Availability

The datasets and code used in this study are available from the authors upon reasonable request.

## Footnotes

* Our primary focus is on the general population and the variance within it, encompassing the entire spectrum of anxiety levels. We intentionally did not exclude participants with high or low anxiety scores to ensure that we captured the full range of variability, which is crucial for our study objectives. To provide reassurance that individuals with self-reported anxiety diagnoses did not disproportionately affect our results or exhibit a distinct association pattern with BIS, we highlighted these participants on theFigure 3scatterplots.

† It is common for researchers using EGI’s dense-array caps (256 electrodes with minimal setup time) to analyze averages of neighboring electrode clusters to improve the signal-to-noise ratio. However, in our laboratory’s experience, with sufficient data, cluster averages produce results very similar to single electrodes. To prioritize simplicity and reliability in identifying markers of trait anxiety, we follow our laboratory’s standard of analyzing single electrodes, as this approach is not only effective but also provides an easier pathway for potential future diagnostic applications. Notably, signal quality was indirectly enhanced by the full 256 electrodes contributing to the ICA-driven artifact correction procedures.

## References

[IMAG.a.44-b1] Allen , J. J. , Coan , J. A. , & Nazarian , M. ( 2004 ). Issues and assumptions on the road from raw signals to metrics of frontal EEG asymmetry in emotion . Biological Psychology , 67 ( 1–2 ), 183 – 218 . 10.1016/j.biopsycho.2004.03.007 15130531

[IMAG.a.44-b2] Andersen , B. , Moore , R. A. , Venables , L. , & Corr , P. J. ( 2009 ). Electrophysiological correlates of anxious rumination . International Journal of Psychophysiology , 71 ( 2 ), 156 – 169 . 10.1016/j.ijpsycho.2008.09.004 18848849

[IMAG.a.44-b3] Aron , A. R. , Robbins , T. W. , & Poldrack , R. A. ( 2014 ). Inhibition and the right inferior frontal cortex: One decade on . Trends in Cognitive Sciences , 18 ( 4 ), 177 – 185 . 10.1016/J.TICS.2013.12.003 24440116

[IMAG.a.44-b4] Barlow , D. H. ( 2004 ). Anxiety and its disorders: The nature and treatment of anxiety and panic . Guilford Press .

[IMAG.a.44-b5] Barry , R. J. , Clarke , A. R. , Johnstone , S. J. , Magee , C. A. , & Rushby , J. A. ( 2007 ). EEG differences between eyes-closed and eyes-open resting conditions . Clinical Neurophysiology , 118 ( 12 ), 2765 – 2773 . 10.1016/J.CLINPH.2007.07.028 17911042

[IMAG.a.44-b6] Barry , R. J. , De Blasio , F. M . , Fogarty , J. S. , & Clarke , A. R. ( 2020 ). Natural alpha frequency components in resting EEG and their relation to arousal . Clinical Neurophysiology , 131 ( 1 ), 205 – 212 . 10.1016/J.CLINPH.2019.10.018 31812081

[IMAG.a.44-b7] Bennett , M. ( 2022 ). Heart rate variability (HRV), cortisol, and trait anxiety in mid-life adults [Master’s thesis]. Marquette University . https://epublications.marquette.edu/theses_open/691

[IMAG.a.44-b8] Berger , H. ( 1929 ). Über das Elektrenkephalogramm des Menschen [On the human electrocephalogram] . Archiv für Psychiatrie und Nervenkrankheiten , 87 ( 1 ), 527 – 570 . 10.1007/BF01797193

[IMAG.a.44-b9] Bush , G. , Luu , P. , & Posner , M. I. ( 2000 ). Cognitive and emotional influences in anterior cingulate cortex . Trends in Cognitive Sciences , 4 ( 6 ), 215 – 222 . 10.1016/S1364-6613(00)01483-2 10827444

[IMAG.a.44-b10] Campbell-Sills , L. , Liverant , G. I. , & Brown , T. A. ( 2004 ). Psychometric evaluation of the behavioral inhibition/behavioral activation scales in a large sample of outpatients with anxiety and mood disorders . Psychological Assessment , 16 ( 3 ), 244 – 254 . 10.1037/1040-3590.16.3.244 15456380

[IMAG.a.44-b11] Caplan , J. B. , Bottomley , M. , Kang , P. , & Dixon , R. A. ( 2015 ). Distinguishing rhythmic from non-rhythmic brain activity during rest in healthy neurocognitive aging . NeuroImage , 112 , 341 – 352 . 10.1016/j.neuroimage.2015.03.001 25769279 PMC4408255

[IMAG.a.44-b12] Caplan , J. B. , Madsen , J. R. , Raghavachari , S. , & Kahana , M. J. ( 2001 ). Distinct patterns of brain oscillations underlie two basic parameters of human maze learning . Journal of Neurophysiology , 21 , 3175 – 3183 . 10.1152/jn.2001.86.1.368 11431517

[IMAG.a.44-b13] Carver , C. S. , & White , T. L. ( 1994 ). Behavioral inhibition, behavioral activation, and affective responses to impending reward and punishment: The BIS/BAS scales . Journal of Personality and Social Psychology , 67 ( 2 ), 319 – 333 . 10.1037/0022-3514.67.2.319

[IMAG.a.44-b14] Cavanagh , J. F. , Meyer , A. , & Hajcak , G. ( 2017 ). Error-specific cognitive control alterations in generalized anxiety disorder . Biological Psychiatry. Cognitive Neuroscience and Neuroimaging , 2 ( 5 ), 413 – 420 . 10.1016/J.BPSC.2017.01.004 28871288 PMC5580396

[IMAG.a.44-b15] Cavanagh , J. F. , & Shackman , A. J. ( 2015 ). Frontal midline theta reflects anxiety and cognitive control: Meta-analytic evidence . Journal of Physiology Paris , 109 ( 1–3 ), 3 – 15 . 10.1016/j.jphysparis.2014.04.003 24787485 PMC4213310

[IMAG.a.44-b16] Chalmers , J. A. , Quintana , D. S. , Abbott , M. J. , & Kemp , A. H. ( 2014 ). Anxiety disorders are associated with reduced heart rate variability: A meta-analysis . Frontiers in Psychiatry , 5 ( 7 ), 100467 . 10.3389/FPSYT.2014.00080 PMC4092363 25071612

[IMAG.a.44-b17] Coan , J. A. , & Allen , J. J. ( 2003 ). Frontal EEG asymmetry and the behavioral activation and inhibition systems . Psychophysiology , 40 ( 1 ), 106 – 114 . 10.1111/1469-8986.00011 12751808

[IMAG.a.44-b18] Cohen , M. X. ( 2014 ). Analyzing neural time series data: Theory and practice . The MIT Press . 10.7551/MITPRESS/9609.001.0001

[IMAG.a.44-b19] Corr , P. J. ( 2008 ). The reinforcement sensitivity theory of personality . Cambridge University Press . 10.1017/CBO9780511819384

[IMAG.a.44-b20] Davidson , R. J. ( 1992 ). Anterior cerebral asymmetry and the nature of emotion . Brain and Cognition , 20 ( 1 ), 125 – 151 . 10.1016/0278-2626(92)90065-T 1389117

[IMAG.a.44-b21] Delorme , A. , & Makeig , B. ( 2004 ). EEGLAB: An open source toolbox for analysis of single-trial EEG dynamics . Journal of Neuroscience Methods , 134 , 9 – 21 . 10.1016/j.jneumeth.2003.10.009 15102499

[IMAG.a.44-b22] Diamond , A. ( 2013 ). Executive functions . Annual Review of Psychology , 64 , 135 – 168 . 10.1146/annurev-psych-113011-143750 PMC4084861 23020641

[IMAG.a.44-b23] Firth , J. , Standen , B. , Sumich , A. , Fino , E. , & Heym , N. ( 2024 ). The neural correlates of reinforcement sensitivity theory: A systematic review of the frontal asymmetry and spectral power literature . Psychophysiology , 61 ( 9 ), e14594 . 10.1111/PSYP.14594 38693649

[IMAG.a.44-b24] Foxe , J. J. , & Snyder , A. C. ( 2011 ). The role of alpha-band brain oscillations as a sensory suppression mechanism during selective attention . Frontiers in Psychology , 2 , 10747 . 10.3389/fpsyg.2011.00154 PMC3132683 21779269

[IMAG.a.44-b25] Gold , C. , Fachner , J. , & Erkkilä , J. ( 2013 ). Validity and reliability of electroencephalographic frontal alpha asymmetry and frontal midline theta as biomarkers for depression . Scandinavian Journal of Psychology , 54 ( 2 ), 118 – 126 . 10.1111/SJOP.12022 23278257

[IMAG.a.44-b26] Gosling , S. D. , Rentfrow , P. J. , & Swann , W. B. ( 2003 ). A very brief measure of the Big-Five personality domains . Journal of Research in Personality , 37 ( 6 ), 504 – 528 . 10.1016/S0092-6566(03)00046-1

[IMAG.a.44-b27] Gray , J. A. ( 1982 ). Gray’s Neuropsychology of anxiety: An enquiry into the functions of septohippocampal theories . Behavioral and Brain Sciences , 5 ( 3 ), 492 – 493 . 10.1017/S0140525X00013170

[IMAG.a.44-b28] Gray , J. A. , & McNaughton , N. ( 2000 ). The neuropsychology of anxiety: An enquiry into the function of the septo-hippocampal system . Oxford University Press . 10.1093/acprof:oso/9780198522713.001.0001

[IMAG.a.44-b29] Grossmann , A. , & Morlet , J. ( 1985 ). Decomposition of functions into wavelets of constant shape, *and related transforms* . In L. Streit (Ed.), Mathematics + Physics (pp. 135 – 165 ). World Scientific . 10.1142/9789814415125_0004

[IMAG.a.44-b30] Gusnard , D. A. , Akbudak , E. , Shulman , G. L. , & Raichle , M. E. ( 2001 ). Medial prefrontal cortex and self-referential mental activity: Relation to a default mode of brain function . Proceedings of the National Academy of Sciences of the United States of America , 98 ( 7 ), 4259 – 4264 . 10.1073/PNAS.071043098 11259662 PMC31213

[IMAG.a.44-b31] Harmon-Jones , E. , & Allen , J. J. B. ( 1997 ). Behavioral activation sensitivity and resting frontal EEG asymmetry: Covariation of putative indicators related to risk for mood disorders . Journal of Abnormal Psychology , 106 ( 1 ), 159 – 163 . 10.1037//0021-843X.106.1.159 9103728

[IMAG.a.44-b32] He , B. J. ( 2014 ). Scale-free brain activity: Past, present, and future . Trends in Cognitive Sciences , 18 ( 9 ), 480 – 487 . 10.1016/J.TICS.2014.04.003 24788139 PMC4149861

[IMAG.a.44-b33] Heym , N. , Ferguson , E. , & Lawrence , C. ( 2008 ). An evaluation of the relationship between Gray’s revised RST and Eysenck’s PEN: Distinguishing BIS and FFFS in Carver and White’s BIS/BAS scales . Personality and Individual Differences , 45 ( 8 ), 709 – 715 . 10.1016/J.PAID.2008.07.013

[IMAG.a.44-b34] Johnson , S. L. , Turner , R. J. , & Iwata , N. ( 2003 ). BIS/BAS levels and psychiatric disorder: An epidemiological study . Journal of Psychopathology and Behavioral Assessment , 25 ( 1 ), 25 – 36 . 10.1023/A:1022247919288

[IMAG.a.44-b35] Jung , T.-P. , Makeig , S. , Humphries , C. , Lee , T.-W. , McKeown , M. J. , Iragui , V. , & Sejnowski , T. J. ( 2000 ). Removing electroencephalographic artifacts by blind source separation . Psychophysiology , 37 , 163 – 178 . 10.1111/1469-8986.3720163 10731767

[IMAG.a.44-b36] Kass , R. E. , & Raftery , A. E. ( 1995 ). Bayes factors . Journal of the American Statistical Association , 90 ( 430 ), 773 – 795 . 10.1080/01621459.1995.10476572

[IMAG.a.44-b37] Klimesch , W. ( 1999 ). EEG alpha and theta oscillations reflect cognitive and memory performance: A review and analysis . Brain Research Reviews , 29 ( 2–3 ), 169 – 195 . 10.1016/S0165-0173(98)00056-3 10209231

[IMAG.a.44-b38] Klimesch , W. , Sauseng , P. , & Hanslmayr , S. ( 2007 ). EEG alpha oscillations: The inhibition-timing hypothesis . Brain Research Reviews , 53 ( 1 ), 63 – 88 . 10.1016/J.BRAINRESREV.2006.06.003 16887192

[IMAG.a.44-b39] Knyazev , G. G. , Savostyanov , A. N. , & Levin , E. A. ( 2004 ). Alpha oscillations as a correlate of trait anxiety . International Journal of Psychophysiology , 53 ( 2 ), 147 – 160 . 10.1016/j.ijpsycho.2004.03.001 15210292

[IMAG.a.44-b40] Knyazev , G. G. , Slobodskaya , H. R. , Safronova , M. V. , Sorokin , O. V. , Goodman , R. , & Wilson , G. D. ( 2003 ). Personality, psychopathology and brain oscillations . Personality and Individual Differences , 35 ( 6 ), 1331 – 1349 . 10.1016/S0191-8869(02)00353-7

[IMAG.a.44-b41] Knyazev , G. G. , Slobodskaya , H. R. , & Wilson , G. D. ( 2002 ). Psychophysiological correlates of behavioural inhibition and activation . Personality and Individual Differences , 33 ( 4 ), 647 – 660 . 10.1016/S0191-8869(01)00180-5

[IMAG.a.44-b42] Kołodziej , A. , Magnuski , M. , Ruban , A. , & Brzezicka , A. ( 2021 ). No relationship between frontal alpha asymmetry and depressive disorders in a multiverse analysis of five studies . eLife , 10 , e60595 . 10.7554/ELIFE.60595 34037520 PMC8154036

[IMAG.a.44-b43] Kopanska , M. , Ochojska , D. , Trojniak , J. , Sarzynska , I. , & Szczygielski , J. ( 2024 ). The role of quantitative electroencephalography in diagnostic workup of mental disorders . Journal of Physiology and Pharmacology: An official journal of the Polish Physiological Society , 75 ( 4 ). 10.26402/JPP.2024.4.02 39415522

[IMAG.a.44-b44] Kreitchmann , R. S. , Abad , F. J. , Ponsoda , V. , Nieto , M. D. , & Morillo , D. ( 2019 ). Controlling for response biases in self-report scales: Forced-choice vs. psychometric modeling of Likert items . Frontiers in Psychology , 10 , 474933 . 10.3389/FPSYG.2019.02309 PMC6803422 31681103

[IMAG.a.44-b45] Kropotov , J. D. ( 2009 ). Quantitative EEG, event-related potentials and neurotherapy (pp. 1 – 542 ). Academic Press . 10.1016/B978-0-12-374512-5.X0001-1

[IMAG.a.44-b46] Li , X. , Zhang , C. , Tan , J. , Ding , L. , Wang , C. , Wang , M. , & Lin , Y. ( 2022 ). Clinical effects of continuous theta burst stimulation for generalized anxiety disorder and a mechanism involving α oscillations: A randomized controlled trial . Journal of Psychiatry and Neuroscience , 47 ( 2 ), E123 – E133 . 10.1503/JPN.210134 35361700 PMC8979658

[IMAG.a.44-b47] McLoughlin , G. , Gyurkovics , M. , Palmer , J. , & Makeig , S. ( 2022 ). Midfrontal theta activity in psychiatric illness: An index of cognitive vulnerabilities across disorders . Biological Psychiatry , 91 ( 2 ), 173 – 182 . 10.1016/J.BIOPSYCH.2021.08.020 34756560

[IMAG.a.44-b48] Menon , V. ( 2023 ). 20 years of the default mode network: A review and synthesis . Neuron , 111 ( 16 ), 2469 – 2487 . 10.1016/J.NEURON.2023.04.023 37167968 PMC10524518

[IMAG.a.44-b49] Metzen , D. , Genç , E. , Getzmann , S. , Larra , M. F. , Wascher , E. , & Ocklenburg , S. ( 2022 ). Frontal and parietal EEG alpha asymmetry: A large-scale investigation of short-term reliability on distinct EEG systems . Brain Structure and Function , 227 ( 2 ), 725 – 740 . 10.1007/S00429-021-02399-1 34676455 PMC8843903

[IMAG.a.44-b50] Mizuki , Y. , Kajimura , N. , Nishikori , S. , Imaizumi , J. , & Yamada , M. ( 1984 ). Appearance of frontal midline theta rhythm and personality traits . Psychiatry and Clinical Neurosciences , 38 ( 4 ), 451 – 458 . 10.1111/j.1440-1819.1984.tb00794.x 6535746

[IMAG.a.44-b51] Mizuki , Y. , Suetsugi , M. , Imai , T. , Kai , S. , Kajimura , N. , & Yamada , M. ( 1989 ). A physiological marker for assessing anxiety level in humans: frontal midline theta activity . Psychiatry and Clinical Neurosciences , 43 ( 4 ), 619 – 626 . 10.1111/j.1440-1819.1989.tb03096.x 2637388

[IMAG.a.44-b52] Moore , R. A. , Gale , A. , Morris , P. H. , & Forrester , D. ( 2006 ). Theta phase locking across the neocortex reflects cortico-hippocampal recursive communication during goal conflict resolution . International Journal of Psychophysiology , 60 ( 3 ), 260 – 273 . 10.1016/J.IJPSYCHO.2005.06.003 16168505

[IMAG.a.44-b53] Näpflin , M. , Wildi , M. , & Sarnthein , J. ( 2007 ). Test–retest reliability of resting EEG spectra validates a statistical signature of persons . Clinical Neurophysiology , 118 ( 11 ), 2519 – 2524 . 10.1016/J.CLINPH.2007.07.022 17892969

[IMAG.a.44-b54] Nash , K. , Gianotti , L. R. , & Knoch , D. ( 2014 ). A neural trait approach to exploring individual differences in social preferences . Frontiers in Behavioral Neuroscience , 8 , 458 . 10.3389/FNBEH.2014.00458 25642176 PMC4295523

[IMAG.a.44-b55] Neo , P. S. , Thurlow , J. K. , & McNaughton , N. ( 2011 ). Stopping, goal-conflict, trait anxiety and frontal rhythmic power in the stop-signal task . Cognitive, Affective and Behavioral Neuroscience , 11 ( 4 ), 485 – 493 . 10.3758/s13415-011-0046-x 21647572

[IMAG.a.44-b56] Palva , S. , & Palva , J. M. ( 2007 ). New vistas for alpha-frequency band oscillations . Trends in Neurosciences , 30 ( 4 ), 150 – 158 . 10.1016/J.TINS.2007.02.001 17307258

[IMAG.a.44-b57] Pfurtscheller , G. ( 1992 ). Event-related synchronization (ERS): An electrophysiological correlate of cortical areas at rest . Electroencephalography and Clinical Neurophysiology , 83 ( 1 ), 62 – 69 . 10.1016/0013-4694(92)90133-3 1376667

[IMAG.a.44-b58] Pion-Tonachini , L. , Kreutz-Delgado , K. , & Makeig , S. ( 2019 ). ICLabel: An automated electroencephalographic independent component classifier, dataset, and website . NeuroImage , 198 , 181 – 197 . 10.1016/J.NEUROIMAGE.2019.05.026 31103785 PMC6592775

[IMAG.a.44-b59] Reznik , S. J. , & Allen , J. J. B. ( 2018 ). Frontal asymmetry as a mediator and moderator of emotion: An updated review . Psychophysiology , 55 , 1 – 32 . 10.1111/psyp.12965 28776710

[IMAG.a.44-b60] Shadli , S. M. , Ando , L. C. , McIntosh , J. , Lodhia , V. , Russell , B. R. , Kirk , I. J. , Glue , P. , & McNaughton , N. ( 2021 ). Right frontal anxiolytic-sensitive EEG ‘theta’ rhythm in the stop-signal task is a theory-based anxiety disorder biomarker . Scientific Reports , 11 ( 1 ), 1 – 12 . 10.1038/s41598-021-99374-x 34611294 PMC8492763

[IMAG.a.44-b61] Shadli , S. M. , High , O. , Byers , B. , Gibbs , P. , Steller , R. , Glue , P. , & McNaughton , N. ( 2020 ). Human anxiety-specific “theta” occurs with selective stopping and localizes to right inferior frontal gyrus . Behavioral Neuroscience , 134 ( 6 ), 547 – 555 . 10.1037/BNE0000316 31219262

[IMAG.a.44-b62] Shlesinger , M. F. , & West , B. J. ( 1988 ). 1/f versus 1/f α Noise . In H. E. Stanley & N. Ostrowsky (Eds.), Random fluctuations and pattern growth: Experiments and models (pp. 320 – 324 ). Springer . 10.1007/978-94-009-2653-0_45

[IMAG.a.44-b63] Smits , D. J. , & Boeck , P. D. ( 2006 ). From BIS/BAS to the big five . European Journal of Personality , 20 ( 4 ), 255 – 270 . 10.1002/PER.583

[IMAG.a.44-b64] Spielberger , C. D. , Gorsuch , R. L. , Lushene , R. , Vagg , P. R. , & Jacobs , G. A. ( 1983 ). Manual for the State-Trait Anxiety Inventory . Consulting Psychologists Press .

[IMAG.a.44-b65] Suetsugi , M. , Mizuki , Y. , Ushijima , I. , Kobayashi , T. , Tsuchiya , K. , Aoki , T. , & Watanabe , Y. ( 2000 ). Appearance of frontal midline theta activity in patients with generalized anxiety disorder . Neuropsychobiology , 41 ( 2 ), 108 – 112 . 10.1159/000026641 10644932

[IMAG.a.44-b66] Sutton , S. K. , & Davidson , R. J. ( 1997 ). Prefrontal brain asymmetry: A biological substrate of the behavioral approach and inhibition systems . Psychological Science , 8 ( 3 ), 204 – 210 . 10.1111/J.1467-9280.1997.TB00413.X

[IMAG.a.44-b67] Tagliazucchi , E. , & Laufs , H. ( 2014 ). Decoding wakefulness levels from typical fMRI resting-state data reveals reliable drifts between wakefulness and sleep . Neuron , 82 ( 3 ), 695 – 708 . 10.1016/J.NEURON.2014.03.020 24811386

[IMAG.a.44-b68] Wall , A. D. , & Lee , E. B. ( 2022 ). What do anxiety scales really measure? An item content analysis of self-report measures of anxiety . Journal of Psychopathology and Behavioral Assessment , 44 ( 4 ), 1148 – 1157 . 10.1007/S10862-022-09973-9

[IMAG.a.44-b69] Whitten , T. A. , Hughes , A. M. , Dickson , C. T. , & Caplan , J. B. ( 2011 ). A better oscillation detection method robustly extracts EEG rhythms across brain state changes: The human alpha rhythm as a test case . NeuroImage , 54 , 860 – 874 . 10.1016/j.neuroimage.2010.08.064 20807577

[IMAG.a.44-b70] Xing , M. , Tadayonnejad , R. , MacNamara , A. , Ajilore , J., Olusola and DiGangi, Phan , K. L. , Leow , A. , & Klumpp , H. ( 2016 ). Resting-state theta band connectivity and graph analysis in generalized social anxiety disorder . NeuroImage. Clinical , 13 , 24 – 32 . 10.1016/J.NICL.2016.11.009 27920976 PMC5126152

[IMAG.a.44-b71] Zhang , Y. , Lei , L. , Liu , Z. , Gao , M. , Liu , Z. , Sun , N. , Yang , C. , Zhang , A. , Wang , Y. , & Zhang , K. ( 2022 ). Theta oscillations: A rhythm difference comparison between major depressive disorder and anxiety disorder . Frontiers in Psychiatry , 13 , 827536 . 10.3389/fpsyt.2022.827536 35990051 PMC9381950

[IMAG.a.44-b72] Zorn , J. V. , Schür , R. R. , Boks , M. P. , Kahn , R. S. , Joëls , M. , & Vinkers , C. H. ( 2017 ). Cortisol stress reactivity across psychiatric disorders: A systematic review and meta-analysis . Psychoneuroendocrinology , 77 , 25 – 36 . 10.1016/J.PSYNEUEN.2016.11.036 28012291

